# Anomaly-Based Intrusion Detection for IoT Microcontrollers Using Power Side-Channel Fingerprinting

**DOI:** 10.3390/s26185769

**Published:** 2026-09-11

**Authors:** Samar Mohamed Hussein Shukry, Frank Kargl, Tallal El-Shabrawy, Amr Talaat Abdel-Hamid

**Affiliations:** 1 Department of Electronics and Networks Engineering, German University in Cairo, New Cairo 11835, Egypt; 2 Institute of Distributed Systems, Ulm University, 89077 Ulm, Germany

**Keywords:** intrusion detection, hardware fingerprinting, power side-channel analysis, deep metric learning, batch-identical microcontrollers, open-set recognition, supply-chain security, temporal stability, IoT security, COTS devices

## Abstract

This study introduces a unified evaluation framework for device-level intrusion detection using power side-channel fingerprints under an open-set threat model. We target post-enrollment device substitution: an adversary replaces a legitimate Commercial Off-The-Shelf (COTS) node with a counterfeit of the same model and firmware after enrollment, during deployment or maintenance, making the substitution invisible to credential-based authentication. To address this scenario, we evaluate seven ESP32-WROOM-32 and seven Arduino Uno R3 devices across four sessions spanning 45 days. Each platform group is drawn from a single manufacturing batch so that measured differences reflect within-batch variation; results are correspondingly established at this scale and for this sourcing. Unlike most prior studies that evaluate fingerprinting under isolated assumptions, this study treats intrusion detection as a system-level problem that requires the joint consideration of temporal stability, open-set recognition, and feature representation. We conduct a two-tier evaluation. Tier 1 characterizes device uniqueness and temporal stability using handcrafted statistical features and neural embeddings trained via triplet + Additive-Margin Softmax (AM-Softmax) loss, analyzed via separation ratios, $d^{\prime }$ scores, F1-scores, and statistical power. Tier 2 applies embeddings to an open-set intrusion-detection protocol using percentile-based thresholding. This analysis reveals that temporal viability is strongly influenced by feature representation under the evaluated conditions. Over 45 days, under an identical Leave-One-Run-Out protocol and scoring rule, neural embeddings achieve a 93.9% true positive rate at a 9.9% false acceptance rate, whereas the same rule applied to handcrafted statistical features yields 66.4% and 35.8%. When each device identity is withheld from training altogether, embeddings hold a 26.0% false acceptance rate against 39.0% for statistical features. Platform comparison shows that ESP32 achieves a 1.80× higher separation ratio than Arduino (3.06 vs. 1.70), with architecture-dependent discriminability. Statistical power analysis yields 89.1% power at d=0.774. These results identify representation-level robustness as a key requirement for practical power-based intrusion detection, while longer-term validation remains an important direction for future work.

## 1. Introduction

**Figure 1:**
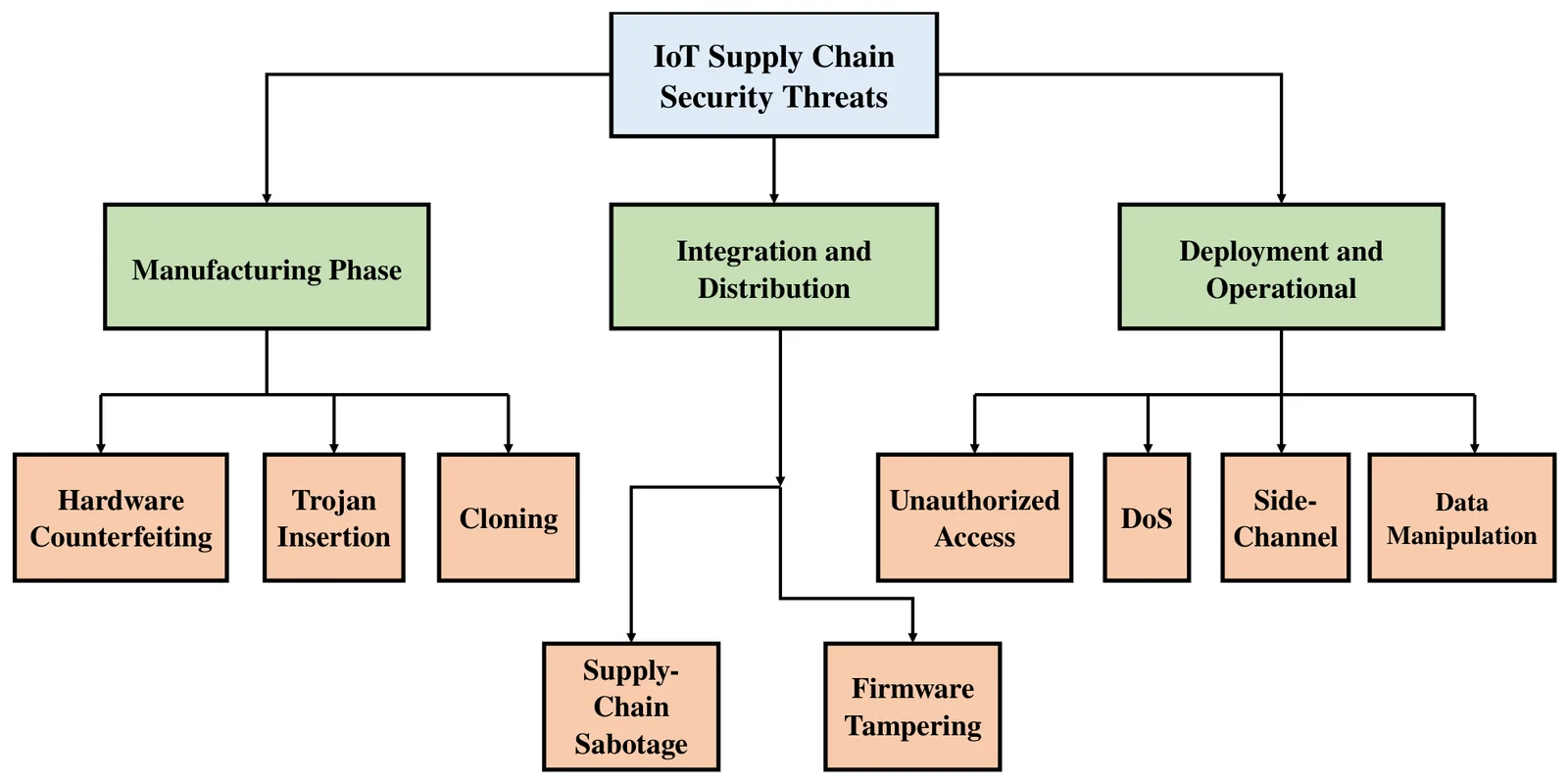
IoT supply-chain security threats across manufacturing, integration, and operational phases.

Internet-of-Things (IoT) networks have expanded rapidly, connecting numerous devices across industrial control, healthcare, transportation, and smart grid networks. While this growth brings efficiency and automation, it also introduces broad and complex security challenges. For instance, attackers exploit supply-chain vulnerabilities that compromise devices during manufacturing or distribution, enabling them to bypass network defenses once systems are deployed [1]. Hence, traditional countermeasures might be insufficient for protecting large-scale IoT environments. As shown in Figure 1, these attack vectors can be grouped into three supply-chain phases:

- **Manufacturing Phase:** hardware counterfeiting, Trojan insertion, and malicious cloning.
- **Integration and Distribution Phase:** supply-chain sabotage and firmware tampering.
- **Deployment and Operational Phase:** unauthorized access, Denial-of-Service (DoS) attacks, side-channel exploitation, and data manipulation.

Recent studies indicate that compromised IoT devices are frequently used as platforms to support large-scale Distributed Denial-of-Service (DDoS) and botnet activity. Such compromises often originate at the device level and can remain unnoticed when malicious hardware operates alongside legitimate devices within the same system. A particularly challenging scenario for intrusion detection arises when adversaries replace or impersonate devices using hardware with identical configurations and valid credentials. These credentials may be reused, copied during provisioning, or introduced through supply-chain or maintenance-stage compromise without requiring direct extraction from deployed devices. This allows attackers to bypass conventional authentication mechanisms, which verify credentials rather than intrinsic hardware properties.

While compromised devices may later be leveraged to support external attacks such as DDoS operations, the more immediate concern for IoT operators is system integrity. Counterfeit or modified devices embedded within operational environments can behave incorrectly under specific conditions, interfere with normal system behavior, or gradually degrade system reliability. These risks motivate intrusion detection mechanisms that operate at the device level and remain effective even when hardware appears functionally identical and valid credentials are present.

To address device impersonation and unauthorized replacement attacks, hardware fingerprinting has emerged as a promising defense mechanism [1,2,3]. It exploits intrinsic physical characteristics of devices to establish unique, unclonable identities [4,5]. Unlike credential-based methods, which rely on cryptographic keys that can be copied or extracted [6,7], hardware fingerprints derive from physical manufacturing imperfections that are inherently difficult to replicate [5,8]. Even devices from the same production line exhibit variations in dopant concentration, gate oxide thickness, and interconnect geometry [4,9], creating device-specific signatures [10,11,12] that prior work has found to remain measurable across repeated acquisitions, although their stability over time has been reported to depend on operating conditions [11].

Several hardware fingerprinting techniques have been proposed in recent literature [3]. One well-established class of hardware-based identification mechanisms is Physical Unclonable Functions (PUFs), which exploit manufacturing-induced variations in integrated circuits to generate device-specific responses and are commonly used for hardware authentication [5,8,9]. PUF-based approaches typically rely on dedicated circuit structures and controlled challenge–response protocols, which limits their applicability for continuous monitoring in deployed systems.

Beyond PUFs, other techniques infer device identity from observable operational characteristics. Radio Frequency (RF) fingerprinting exploits variations in wireless signal characteristics arising from analog component tolerances [13,14,15], while timing-based fingerprinting leverages clock skew and execution latency differences [16]. However, RF methods require specialized Software-Defined Radio (SDR) equipment and are susceptible to environmental interference [13,15], while timing-based methods demand precise synchronization and are vulnerable to temperature drift [16].

In contrast to these approaches, power side-channel fingerprinting offers several practical advantages in deployment scenarios where device-level power monitoring is available. Unlike RF-based approaches that rely on remote over-the-air observations or timing-based methods that require precise synchronization, power analysis operates directly on current measurements taken at the device power supply. Moreover, power signatures reflect the underlying hardware characteristics, enabling detection of hardware replacement through signature mismatch. While power measurements require physical access to the device, this approach integrates naturally into existing on-device or in-line monitoring setups without modifying device hardware, making it well suited for IoT deployments where devices can be instrumented during installation or maintenance.

Our proposed power side-channel fingerprinting framework builds on this concept to detect hardware replacement in batch-identical IoT devices under controlled execution conditions. Using simple shunt-resistor measurements, we record the shunt voltage, which is proportional to the supply current (Section 4.2), while devices execute a dedicated, deterministic measurement workload designed to minimize variability from external inputs. This workload produces power traces that primarily reflect manufacturing-induced characteristics, such as transistor switching delays, gate-level mismatches, and circuit parasitics, resulting in subtle but consistent differences that remain unique to each device.

**Figure 2:**
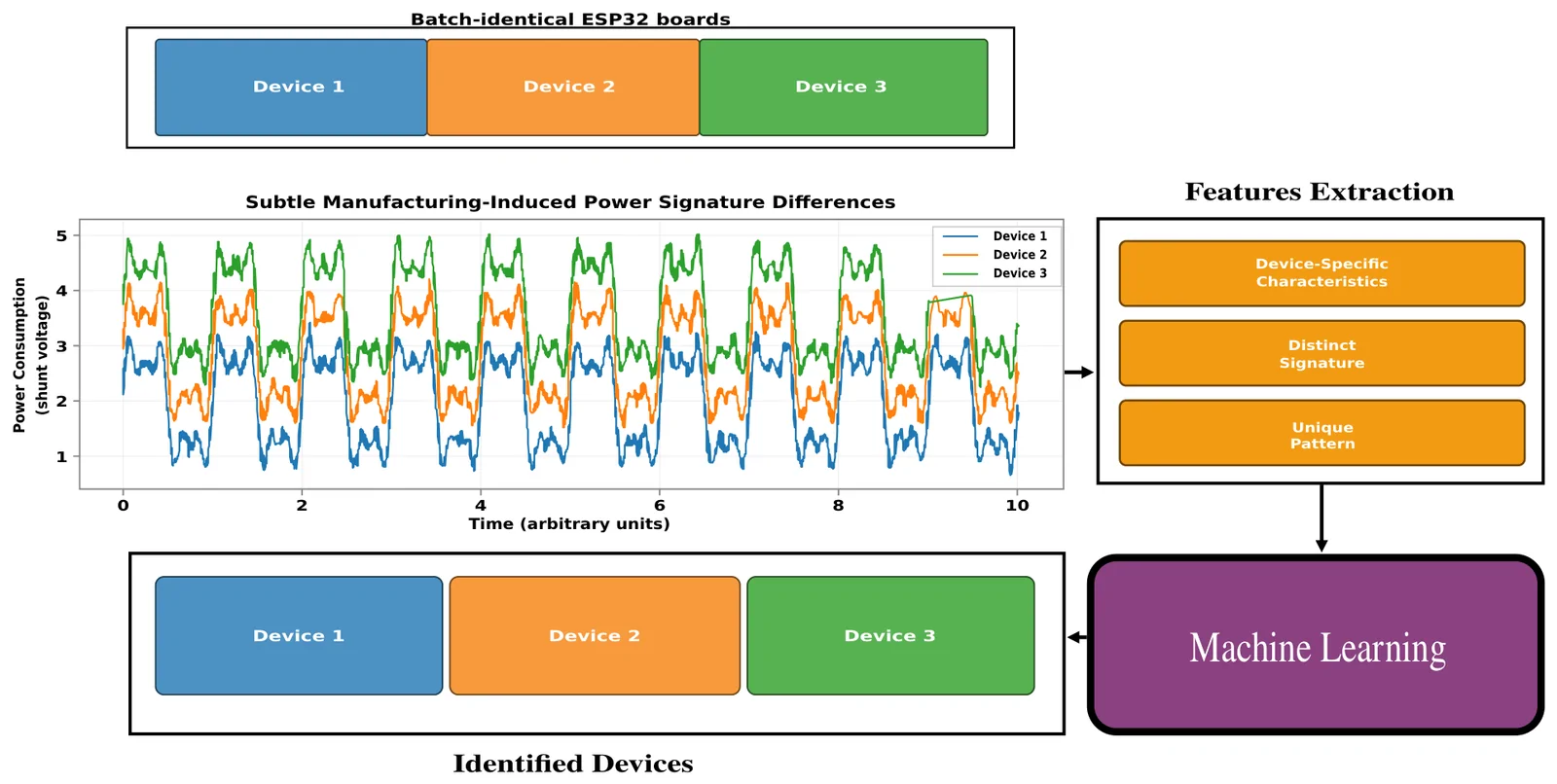
Conceptual overview of the proposed power side-channel fingerprinting framework.

To assess how different hardware subsystems contribute to fingerprint quality, the framework employs multiple predefined measurement workloads that deterministically stress compute logic, peripheral access, and communication subsystems. Device identity verification is performed by re-executing the same predefined measurement workload used during enrollment and comparing the resulting power signature against the stored reference. By constraining execution paths and inputs, this approach enables reproducible power measurements while capturing hardware-specific characteristics and stable aspects of software execution. Under this model, the system can distinguish legitimate devices from batch-identical hardware clones. Because the recorded signature also reflects the executed instruction stream, modifications to the measurement workload’s firmware would in principle perturb it as well. Figure 2 illustrates the overall concept: manufacturing-induced variations in power consumption are converted into device-specific features, which are then analyzed using machine learning models for intrusion detection.

While power side-channel fingerprinting provides a promising mechanism for device-level intrusion detection, its effectiveness depends critically on the stability of power signatures over time. Manufacturing-induced variations are expected to persist, yet measured power traces can still be influenced by environmental factors such as temperature, supply voltage fluctuations, and operational stress, even under controlled laboratory conditions. In addition, component aging and repeated execution cycles may gradually affect electrical behavior. As a result, the temporal consistency of power side-channel fingerprints—and their suitability for reliable intrusion detection beyond a single measurement session—requires careful empirical evaluation. This issue is particularly relevant for IoT deployments, where devices are expected to operate continuously over extended periods under variable environmental conditions and re-enrollment or frequent recalibration may not be practical [1].

This work investigates the feasibility of using power side-channel fingerprints as a stable basis for device-level intrusion detection in batch-identical IoT microcontrollers. Rather than proposing power side-channel fingerprinting as a standalone authentication mechanism, our goal is to characterize its discriminative capability and temporal behavior under controlled execution conditions. Specifically, three key properties are studied: (i) the ability of power side-channel fingerprints to distinguish batch-identical devices executing identical workloads, (ii) the stability of these fingerprints across multiple measurement sessions spanning 45 days, and (iii) the impact of feature representation—handcrafted statistical features versus learned neural embeddings—on temporal robustness. To support intrusion detection requirements, we further evaluate an open-set recognition protocol that enables rejection of previously unseen devices. Empirical insight is provided into the strengths and limitations of power side-channel fingerprinting as a practical component of IoT Intrusion Detection Systems.

Power side-channel fingerprints are sensitive to both hardware identity and software execution behavior, since the power trace reflects the interaction between manufacturing-specific circuit characteristics and the executed workload. Our experimental design exploits this dual sensitivity: cross-device comparisons isolate hardware-level variations, while cross workload comparisons confirm execution-dependent encoding, and together they establish that the recorded signature carries both the identity of the device and the identity of the executed workload.

A further challenge is that ESP32 and Arduino Uno consume power in the milliwatt range, where manufacturing-induced differences are substantially smaller than those reported for desktop PCs [11] or high-power equipment [12,17]. The resulting lower signal-to-noise ratios make fingerprint extraction inherently more difficult than in prior work.

As summarized in Table 1, no existing study in our comparison satisfies more than two of the six criteria necessary for deployment-aware evaluation, and none combines power-based measurement with microcontroller-class hardware, as detailed in Section 2. This work defines and experimentally validates a unified evaluation framework for power-based intrusion detection on batch-identical low-power IoT microcontrollers. The specific contributions are detailed in Section 2. A key empirical finding enabled by this unified evaluation is that temporal viability of power side-channel fingerprints is strongly influenced by feature representation: learned embeddings preserve device separability across sessions where handcrafted statistical features degrade severely. To the best of our knowledge, no prior work simultaneously satisfies all six dimensions necessary for power-based intrusion detection: (i) power-based measurement, (ii) MCU-level operation, (iii) same-model device discrimination, (iv) open-set rejection of unknown devices, (v) multi-session temporal evaluation, and (vi) intrusion detection framing. This gap motivates the unified evaluation framework presented in this study.

The remainder of this paper is organized as follows. Section 2 reviews related work and validates the identified research gaps. Section 3 defines the threat model and describes the proposed framework and methodology. Section 4 explains the experimental setup and evaluation conditions. Section 5 presents and analyzes the results, including single-run comparisons, multi-run temporal analysis, and neural embedding performance. Section 6 discusses implications and future research directions, and Section 7 concludes the paper.

## 2. Related Work

Prior work on IoT security spans a wide range of device fingerprinting and intrusion detection techniques designed for resource-constrained hardware. This section reviews four main areas of research: power side-channel fingerprinting, RF-based identification, anomaly detection and open-set recognition, and temporal stability. We emphasize power-based methods for separating batch-identical devices, identifying gaps motivating our 45-day evaluation on ESP32 microcontrollers.

### 2.1. Power-Based IoT Fingerprinting

Power consumption analysis offers non-invasive hardware differentiation by leveraging manufacturing process variations, though existing approaches vary significantly in their technical foundations, application domains, and inherent limitations. A comprehensive understanding of these differences is essential to position our contributions clearly.

Spolaor et al. [10] proposed PowerID, which fingerprints USB peripherals from the power they draw over the bus. Evaluated on a dataset of 82 peripherals spanning 35 models and eight device types, PowerID recognizes a peripheral’s type, model, activity, and individual identity and therefore separates devices of the same model. Its representation, however, is a large handcrafted statistical feature set computed directly from raw power segments, and its evaluation is confined to a single acquisition campaign on bus-powered peripherals. Temporal stability across separated sessions, rejection of non-enrolled devices, and operation on microcontroller-class hardware all fall outside its scope. Whether feature sets of this kind retain their discriminative power once enrollment and verification are separated in time is precisely the question our controlled comparison of statistical features against learned embeddings addresses.

The one existing study that reaches microcontroller-class hardware does so through a different physical channel, and is included here because its target platform—rather than its modality—is what makes it the closest predecessor. Xiao et al. [2] introduced MCU-Token, which binds a hardware fingerprint to each request so that it can serve as an access token resistant to token compromise. The fingerprint is assembled from six on-chip responses—digital-to-analog and analog-to-digital conversion bias, floating-point unit behavior, pulse-width-modulation accumulation, real-time-clock frequency and phase, and SRAM start-up state—rather than from an externally measured supply current, making it a self-measurement of peripheral behavior rather than a power side-channel. Across 60 boards spanning three families (ESP32-S2, STM32F103 and STM32F429) they report 97.78% TPR at 4.87% FPR, and they examine robustness to ambient temperature and humidity. What their evaluation does not address is fingerprint behavior across measurement sessions separated in time, rejection of devices that were never enrolled, or an intrusion-detection framing with an explicit adversary model. The fingerprint is also read by the device’s own firmware, so its integrity rests on that firmware remaining uncompromised—an assumption an externally measured supply current does not require.

A second line of work brings power side-channel analysis into an operational intrusion-detection setting. Myridakis et al. [19] established the minimal viable form on a deployed IP camera, correlating supply current recovered through a series sensing element with the device’s functional state to flag operational anomalies, without modifying the device or requiring cooperation from its firmware. Jung et al. [21] scaled this into DeepAuditor, an online system for multiple IoT devices built around a dedicated in-line measurement unit and a distributed Convolutional Neural Network (CNN) classifier with encrypted inference. Lightbody et al. [22] released Dragon_Pi, a dataset of normal and under-attack power traces from DragonBoard 410c and Raspberry Pi testbeds, together with Dragon_Slice, an unsupervised convolutional autoencoder trained on normal traces alone that reaches an area under the curve of up to 0.89 after post-processing.

These systems share a structural assumption that bounds what they can detect. Each scores the behavior of a device whose identity is presumed correct: device identity is never estimated, non-enrolled hardware is never rejected, and evaluation is confined to a single acquisition campaign. Dragon_Slice makes the consequence clearest—trained only on legitimate behavior, it generalizes to attacks it has never seen, yet a substituted but functionally identical board running the same firmware produces normal-looking traces and remains invisible to it. That substitution scenario is what motivates the device-identity formulation adopted here.

Beyond microcontroller-focused studies, power and current consumption have been used for device fingerprinting on higher-power platforms. Botvinnik et al. [11] profiled individual assembly instructions on 291 desktop and server systems, achieving high identification accuracy but reporting significant sensitivity to temperature changes. Ronkin et al. [17] achieved 100% identification precision across five identical electrical devices using externally measured current, and in a follow-up study [12] extended this to 40 same-model displays at approximately 94% precision. Park et al. [20] combined power with memory access side-channels for anomaly detection, though only on emulated hardware. These studies demonstrate the potential of power-based differentiation but remain limited to high-power platforms, small device pools, or single-session evaluation—none operates on low-power Commercial Off-The-Shelf (COTS) microcontrollers with multi-session temporal analysis.

In adjacent domains, behavioral fingerprinting approaches [23,24] use CPU, GPU, and RAM behavior to distinguish identical single-board computers, while Huang et al. [25] combined an additive angular margin Softmax objective with an OpenMax-style rejection layer for open-set IoT device authentication, evaluated on both simulated signals and real Automatic Dependent Surveillance-Broadcast (ADS-B) recordings. These works confirm that manufacturing variations are observable through multiple channels, but they do not employ power side-channels and lack temporal or open-set evaluation in the power domain.

These studies reveal three critical gaps for power-based MCU fingerprinting: (1) no multi-session temporal evaluation, (2) closed-set assumptions preventing counterfeit detection, and (3) no controlled comparison of statistical versus learned features under identical temporal conditions. These gaps are analyzed systematically in Section 2.5.

### 2.2. RF-Based Fingerprinting Approaches

RF fingerprinting exploits transmitter hardware imperfections at the physical layer, and its literature has converged on two failure modes that are directly instructive here.

The first is temporal. Elmaghbub and Hamdaoui [3] developed a domain-agnostic CNN identifier for Zero-Trust IoT security that exceeds 99% accuracy within a single domain but falls to 93% across days and 95% across locations. Alhazbi et al. [26] isolated the same phenomenon as the “day-after-tomorrow” effect, showing that accuracy collapses from close to 1.0 to roughly 0.5 once the radios are power-cycled between the training and testing captures and that the variability of the wireless channel alone does not account for the loss. Jagannath et al. [15] report accuracy gains of up to 50.5% from an attentional multi-task architecture evaluated on real WiFi and Bluetooth emissions captured months apart, indicating that the degradation is addressable by representation design rather than intrinsic to the modality—an observation that parallels the finding reported in Section 5.2.

The second is instrumental. Shen et al. [13] showed that the receiver hardware is an equally strong confound, recovering up to 20% accuracy through collaborative inference and up to 40% for under-performing receivers through fine-tuning, across ten LoRa transmitters and twenty software-defined-radio receivers, while Lomba et al. [14] respond to the same instability by selecting and tuning the fingerprinting technique adaptively for Industrial IoT authentication.

RF fingerprinting’s key limitation for intrusion detection contexts is its requirement for specialized Software-Defined Radio equipment, susceptibility to multipath interference and channel variability, and inability to operate on wired or non-transmitting devices. In contrast, power-based approaches require only current measurement capabilities already present in many IoT systems, directly reflecting device-level electrical characteristics without environmental interference.

### 2.3. Anomaly Detection and Open-Set Recognition

Conventional Intrusion Detection Systems assume closed-set operation, limiting effectiveness against zero-day attacks. Surveys of machine- and deep-learning based IDS for IoT report strong detection performance on the attack classes represented during training [27], whereas reviews devoted specifically to zero-day detection identify generalization to previously unseen attack patterns as the principal unresolved weakness of these frameworks [28]. This gap motivates physical-layer alternatives.

Tax and Duin [29] introduced Support Vector Data Description (SVDD), a one-class classification method learning a hypersphere boundary around normal training data. SVDD is effective for anomaly detection but requires representative training data and struggles with high-dimensional feature spaces common in IoT environments. Open-set recognition tackles the closed-set assumption itself: Bendale and Boult [30] introduced OpenMax, a calibrated rejection layer that allows a deep classifier to abstain on inputs drawn from classes absent at training time, and Huang et al. [25] adapted this idea to IoT device authentication from radio signals.

What these approaches share with network-level detection is the representation they operate on. Recent graph-based network IDSs such as that of Villegas-Ch et al. [31] model IoT network traffic as a dynamic graph and apply graph neural networks to detect distributed attacks, achieving a 0.95 F1-score and 0.98 Area Under the Receiver Operating Characteristic Curve (AUC-ROC) on Denial-of-Service, spoofing, and man-in-the-middle scenarios. In every case, the observable—a radio signal, a traffic pattern, a classifier logit—is one that a substitute device reproduces faithfully once it carries the same firmware and credentials. None can therefore distinguish batch-identical hardware clones, which is why the rejection mechanism has to be attached to a physical-layer observable rather than a behavioral one.

Applying open-set concepts to power-based hardware fingerprinting requires addressing feature stability over time, computational constraints, and distinguishing manufacturing variations from environmental noise.

### 2.4. Manufacturing Variations and Temporal Stability

Physical Unclonable Functions (PUFs) exploit manufacturing variations for device authentication using dedicated circuit structures. Suh and Devadas [5] introduced ring-oscillator PUFs for device authentication and secret key generation, showing that intra-die delay variations alone yield chip-unique responses, although the construction requires dedicated on-chip circuitry and a controlled challenge–response interface. Rührmair et al. [9] subsequently demonstrated, on both simulated and silicon challenge–response data, that arbiter, XOR-arbiter, lightweight-secure and feed-forward PUFs can be cloned numerically by machine learning, bounding the security such constructions provide. Kang et al. [8] addressed the complementary problem of PUF response instability through fuzzy extractors, though the error-correcting machinery involved consumes resources that limit deployment on minimalist IoT microcontrollers. Memory-based variants such as SRAM PUFs avoid dedicated delay circuits, but require controlled power cycling and dedicated access protocols that conflict with normal operation.

Anagnostopoulos et al. [32] demonstrated NAND Flash PUFs on COTS hardware, but voltage variations degrade robustness significantly and operational overheads (read/write latencies, wear leveling sensitivity) limit applicability for continuous intrusion detection. In contrast, power side-channel fingerprinting operates on current measurements already available in many IoT systems, as described in Section 1, making it more suitable for continuous device verification in COTS infrastructure.

### 2.5. Comparative Analysis and Research Gaps

Table 1 synthesizes the preceding review by evaluating power and current-based fingerprinting studies against six requirements for practical IoT intrusion detection: use of power or current measurements (Power), operation on microcontroller-class hardware (MCU), discrimination of same-model devices (Same-model), ability to reject unknown devices (Open-set), evaluation across multiple temporal sessions (Temporal), and framing as intrusion detection (Intrusion Det.). The table additionally includes MCU-Token [2], which is not power-based but is the closest microcontroller-level hardware-fingerprinting predecessor.

Read across the six dimensions, the studies cluster by what they optimize for. Identification-oriented work [10,11,12,17,18] establishes that manufacturing variation is measurable and that same-model units are separable but remains closed-set and—with the partial exception of Botvinnik et al. [11], whose repeated acquisitions span at most ten days—confined to a single campaign. Detection-oriented work [19,20,21,22] adopts an intrusion-detection framing but characterizes anomalous behavior on a device whose identity is presumed correct, and therefore neither discriminates between same-model units nor rejects hardware it has not seen. No prior row satisfies more than two of the six criteria in full, and same-model discrimination and intrusion-detection framing never co-occur in any of them.

The most consequential observation concerns the microcontroller column. MCU-Token reaches microcontroller-class hardware and discriminates same-model devices but does so through on-chip responses read by the device’s own firmware rather than through power measurement; every study that does measure power operates on desktop, server, mains-powered or single-board platforms. To the best of our knowledge, no prior work fingerprints microcontroller-class devices through externally measured supply current at all, and consequently none addresses open-set rejection or multi-session temporal stability in that regime. This is the intersection the present work targets.

A structured comparison across these dimensions reveals that prior work satisfies only isolated subsets of deployment requirements, leading to fragmented conclusions about fingerprinting effectiveness.

#### 2.5.1. Temporal Stability Evaluation

With the single partial exception of Botvinnik et al. [11], whose repeated acquisitions are separated by at most ten days and already show substantial degradation, every power-based study reviewed here evaluates fingerprints within a single acquisition campaign. No prior work reports multi-session evaluation spanning weeks, and none does so on microcontroller-class hardware.

#### 2.5.2. Open-Set Recognition Capability

Every reviewed study operates in closed-set mode, preventing detection of unknown devices—a critical requirement for intrusion detection.

#### 2.5.3. Statistical Features vs. Neural Network Embeddings

No study compares handcrafted features with learned representations under identical temporal conditions, leaving no empirical guidance for feature selection when temporal robustness is required.

#### 2.5.4. Application Domain Mismatch

Existing work focuses on closed-set identification or malware detection, where all legitimate devices are known during training. These approaches cannot reject unauthorized clones carrying valid credentials, requiring open-set recognition protocols.

#### 2.5.5. Absence of Formal Threat Modeling

To the best of our knowledge, no prior power side-channel fingerprinting study defines a formal adversary model specifying attacker capabilities, constraints, and assumptions about measurement infrastructure. Spolaor et al. [10], Ronkin et al. [12,17], and Botvinnik et al. [11] treat fingerprinting as a classification task without security framing. Jung et al. [21] and Lightbody et al. [22] frame their work as intrusion detection but characterize the attack rather than the attacker, without specifying adversary capabilities or constraints on the measurement chain. Xiao et al. [2] address authentication but assume a closed-set setting without modeling the replacement-with-valid-credentials scenario. Without explicit threat models, it is unclear under what conditions each approach provides security guarantees, making cross-study comparison difficult and deployment guidance unreliable.

Collectively, these five gaps indicate that prior work evaluates fingerprinting performance under fragmented assumptions and without formal security grounding. In contrast, this work treats intrusion detection as a system-level problem that integrates temporal robustness, open-set recognition, same-model discrimination, and explicit threat modeling within a single evaluation framework.

### 2.6. Contributions

This work contributes five specific advances toward power-based intrusion detection.

#### 2.6.1. Design-Space Definition for Power-Based Intrusion Detection

We identify six complementary evaluation dimensions—external power or current measurement, low-power MCU-level operation, same-model discrimination, open-set rejection of unknown devices, multi-session temporal evaluation, and intrusion-detection framing—and, to our knowledge, our framework is the first to address all six within a single study, whereas no prior work satisfies more than two in full (Table 1). The controlled comparison of statistical features against learned embeddings, reported in Section 5.2, is the mechanism through which the temporal dimension is addressed.

#### 2.6.2. Multi-Session Temporal Analysis

We conduct a multi-session power side-channel fingerprinting study—to the best of our knowledge, the first on MCU-class devices—with measurements across 45 days (4 sessions), comparing statistical features and neural embeddings under identical controlled conditions. While this timeframe does not yet constitute long-term validation, it reveals temporal degradation patterns that are not observable in single-session evaluations.

#### 2.6.3. Open-Set Intrusion Detection Framework

We introduce an open-set evaluation protocol enabling unknown device rejection for power side-channel analysis. To the best of our knowledge, this represents the first adaptation of open-set concepts—previously demonstrated in RF and network domains—to power side-channel fingerprinting, addressing the closed-set limitation in existing approaches, though practical deployment readiness requires extensive additional validation.

#### 2.6.4. Controlled Feature-Type Comparison

We systematically compare traditional statistical features with learned neural embeddings under identical experimental conditions across four measurement sessions. As far as we know, this controlled comparison provides the first direct empirical characterization of temporal behavior across feature representations in power side-channel fingerprinting. While generalization to other platforms, environments, and longer time horizons requires further investigation, the results reveal a consistent and previously unreported dependency of temporal robustness on feature representation. Our approach employs a combined triplet loss and AM-Softmax objective to learn device embeddings. We further conduct ablation analysis to isolate the contribution of each component and identify which mechanisms drive temporal robustness (Section 5.5).

#### 2.6.5. Cross-Platform Exploitability Analysis

We compare ESP32 and Arduino microcontrollers under identical workloads and measurement protocols to quantify architecture-dependent fingerprinting feasibility. This platform comparison, absent in existing single-platform studies, shows that fingerprint quality differs substantially between the two ends of the low-cost COTS range.

While further validation across longer time spans and larger device populations is required, these contributions collectively outline a deployment-aware evaluation methodology for power-based intrusion detection.

## 3. Proposed Framework

This section defines the threat model and describes the proposed framework methodology. The methodology comprises three stages: (1) feature extraction and preprocessing, (2) single-run analysis to establish baseline device distinctiveness, and (3) multi-run temporal analysis to examine fingerprint stability and neural embedding effectiveness. Together, these stages assess device separation quality, temporal stability, and open-set intrusion detection feasibility.

### 3.1. Threat Model and Attack Scenarios

This subsection outlines the assumed capabilities and limitations of a potential adversary, describes relevant attack scenarios, and defines the foundational assumptions guiding the experimental evaluation.

#### 3.1.1. Adversary Model

*(a) Capabilities:* The adversary has access to batch-identical COTS microcontrollers sourced from the same manufacturer and production line as those used in legitimate devices, ensuring functional equivalence. The attacker can introduce unauthorized devices during the supply chain or physically replace deployed devices during maintenance periods.

*(b) Constraints:* The adversary is assumed to be unable to

- Tamper with the power monitoring infrastructure. Power monitoring components are typically deployed in physically secured gateway locations, making targeted tampering detectable through routine calibration checks.
- Modify on-board voltage regulation without being detected. Voltage regulator modifications require physical access and specialized equipment, making such alterations evident during visual inspection.
- Synthesize arbitrary power signatures through hardware alterations. Replicating manufacturing-induced power variations requires precise control over transistor-level characteristics that are infeasible to reproduce without semiconductor fabrication capabilities.

*(c) Measurement Infrastructure Assumption:* This threat model assumes that measurement hardware (shunt resistor, Analog-to-Digital Converter (ADC), data acquisition unit), data transmission paths, and enrollment processes are physically protected and operate under organizational control. This assumption is realistic in established IoT deployment classes including industrial Supervisory Control and Data Acquisition (SCADA) environments [33], smart metering deployments [34], and managed enterprise IoT [35]. If partially violated, cross-session consistency checks can flag anomalous readings. Under full compromise, an adversary controlling the measurement chain could defeat the scheme in either direction, by altering or replacing the stored reference signatures so that a counterfeit device matches, or by replaying a previously recorded measurement of the legitimate device. In both cases, the verifier observes a “match” for an illegitimate device and the substitution goes undetected, rendering power side-channel fingerprinting ineffective; this boundary condition is shared by all physical-layer authentication mechanisms [3,5]. Architectural mechanisms that reduce reliance on this assumption in deployed systems are outlined in Section 6.3. Power side-channel fingerprinting is intended as one layer within a defense-in-depth architecture [36]. Its value lies in complementing credential-based and network-layer defenses with a physical-layer verification capability that is resistant to credential theft.

*(d) Objective:* To gain unauthorized access to a trusted IoT network by replacing legitimate devices or introducing unregistered nodes, with the intent to extract data, manipulate sensor outputs, or disrupt system functionality.

#### 3.1.2. Physical Device Replacement Scenario

In this attack scenario, an adversary physically replaces a legitimate microcontroller with another device from the same manufacturing batch. The replacement unit contains identical firmware and cryptographic credentials, making it indistinguishable through traditional authentication mechanisms such as Public Key Infrastructure (PKI) or Message Authentication Code (MAC).

However, the replacement is a distinct physical device whose manufacturing variations produce a distinctive power consumption signature. During the deterministic measurement workload, this signature will differ from the enrolled reference, triggering a fingerprint mismatch. This mismatch alerts operators that a substitution has occurred, prompting further investigation. The approach does not determine whether the replacement is malicious or benign. Our work focuses on detecting physical device substitution through fingerprint mismatches, independent of the replacement device’s internal state or malicious intent.

#### 3.1.3. Evaluation Scope and Assumptions

*(a) Experimental Focus:* We evaluate the temporal consistency of power side-channel fingerprints over 45 days (four sessions) using consistent measurement procedures to assess feasibility for periodic verification protocols. Practical aspects of deployment—such as sensor placement, real-time processing, and alert response mechanisms—are beyond the current scope and are left for future work.

*(b) Trusted Enrollment Assumption:* Our threat model targets in-field device replacement: an adversary physically swaps an enrolled device with a different one during operational deployment or maintenance. To detect such substitutions, we capture the fingerprint baseline while the device is still under organizational control—during installation, commissioning, or initial deployment. This happens before the device enters unsecured environments where substitution becomes possible.

We do not address supply-chain compromises that occur before enrollment. Detecting a compromised device at the enrollment stage would require validating the device’s authenticity before fingerprinting, which falls outside the scope of this approach. In practice, this means organizations need procedures to verify devices when they arrive from the supplier and before recording the baseline fingerprint. These steps help ensure that the enrolled device is legitimate.

*(c) Environmental Conditions:* Measurements were conducted in a standard university laboratory under nominally consistent ambient conditions. Environmental factors such as temperature, humidity, and supply voltage were not continuously monitored or recorded. However, all sessions used the same laboratory, equipment, and measurement protocols, ensuring that any environmental variation affected all devices equally and did not introduce systematic bias between devices within a session. The temporal evaluation is structured around this variation rather than around an assumption that it was absent: each session is in turn withheld from training entirely (Section 3.4.3), so whatever differed between sessions—in ambient conditions, supply, or the measurement chain—is present in the data on which verification is tested. Reported cross-session performance is therefore obtained across that variation. The magnitude of each individual contribution is not resolved by this design, and separating them is addressed in Section 6.4.

### 3.2. Statistical Feature Extraction and Preprocessing

Devices execute standardized workloads (specifications in Section 4.3) that stress different hardware subsystems—computational logic, memory access patterns, and I/O peripherals. We record power traces continuously using the methodology described in Section 4.2. These traces are segmented into fixed-duration windows and characterized using the following 19 statistical features [4] that capture manufacturing variations in transistor switching thresholds, gate delays, and analog circuit characteristics:

- Mean.
- Standard Deviation.
- Median Absolute Deviation (MAD).
- Skewness.
- Spectral Entropy (computed on |rfft(·)|).
- 10th to 90th quantiles.
- Distance between mean and median.
- Mean/Median/Standard Deviation of Sequence of Differences.
- Number of Peaks.

The extracted feature datasets undergo a preprocessing pipeline designed to improve discriminability while preserving statistical integrity. 

#### 3.2.1. Stage 1: Feature Quality Diagnosis (Marking Only)

Each feature is examined over the pooled set of all its values across every device, session and window. A feature is marked for removal if it satisfies any of the following three criteria:

- Fewer than 20 valid observations;
- Fewer than 5 unique values, which indicates quantization-limited or degenerate behavior rather than device behavior;
- Variance below $1\times 10^{-9}$.

A fourth diagnostic—multimodality—is computed for every feature that passes these three tests, but it does not cause removal. Its purpose is to characterize the distribution so that the appropriate downstream summary statistics can be selected. Multimodality is assessed by two independent tests whose evidence is summed: one- and two-component Gaussian Mixture Models are fitted to the pooled values of the feature, and an Akaike Information Criterion difference exceeding 10 in favor of the two-component model contributes 1.0, as does a Bayesian Information Criterion difference exceeding 6; independently, a kernel density estimate is evaluated on a 500-point grid and the detection of more than one peak at a prominence of 5% of the maximum density contributes a further 1.0. A cumulative score of 2.0 or above classifies the feature as multimodal. Multimodal features are deliberately retained: for a workload that alternates between active computation, wireless transmission and a low-power idle phase, more than one mode is the expected signature rather than an artifact, and discarding such features would remove exactly the structure the fingerprint depends on. Stage 1 modifies no data; it emits only a per-feature diagnosis.

Applied to the multi-session ESP32 Networked Sensor Monitoring dataset (seven devices, four sessions, 532 windows in total), Stage 1 marks a single feature. The median absolute deviation is removed for insufficient cardinality: pooled across every device and session, it takes fewer than five distinct values, a quantization consequence of the 10-bit converter, since a median-based statistic computed on integer-valued samples can only take multiples of the converter’s least significant bit. Of the eighteen features retained, seventeen are classified as multimodal and one as unimodal.

#### 3.2.2. Stage 2: Deterministic Removal

Stage 2 performs no assessment of its own. It applies the Stage 1 mask, deleting the marked columns from every run of every device, so that all devices are described by an identical feature set. For this dataset, the representation is reduced from 19 features to 18.

#### 3.2.3. Stage 3: MAD-Based Screening and Post-Clipping Re-Check

Stage 3 identifies features that become numerically degenerate once robust outlier bounds are applied. Robust bounds are computed for each retained feature using the median absolute deviation, which is insensitive to the measurement transients and ADC saturation events that would distort a variance-based bound. For each feature *X*, the bounds are

(1) $$\mathrm{bounds}=\mathrm{median}\left(X\right)\pm 3\cdot \frac{\mathrm{MAD}(X)}{0.6745}$$

where *X* represents the feature vector containing all samples for a given feature, $\mathrm{MAD}\left(X\right)=\mathrm{median}(|X_{i}-\mathrm{median}\left(X\right)|)$ is the median absolute deviation, and the constant 1/0.6745≈1.4826 ensures consistency with the standard deviation under a Gaussian distribution [37]. The resulting bounds are equivalent to $\pm 3\hat{\sigma }$, where $\hat{\sigma }$ is the robust scale estimate, retaining approximately 99.7% of normally distributed samples. Bounds are estimated from the pooled values of each feature across every device and session so that a single common transformation defines the degeneracy test for the whole dataset.

The Stage 1 criteria are then re-applied to the bounded values. This second pass identifies a further descriptor: the median of the sequence of successive differences passes Stage 1 with exactly five distinct values and a variance of $2.45\times 10^{-5}$, but its median absolute deviation is $2.78\times 10^{-17}$, because more than half of all windows share an identical value. Its robust interval accordingly collapses to a single point and the descriptor carries no usable variation, so it is removed. This is the characteristic behavior of a highly discrete quantity under robust bounds, and the re-check is what keeps such a column out of the analysis.

Together with the descriptor removed at Stage 2, this yields the 17-dimensional feature vector that forms the input to the statistical baselines of Section 5 and to the embedding network of Section 3.4.3. The three-stage sequence is applied identically to all datasets in this study.

### 3.3. Single-Run Baseline Evaluation

Our single-run analysis establishes baseline device distinctiveness on the initial (Day 0) measurement session, addressing two fundamental questions: (1) Do manufacturing-induced power variations enable discrimination of batch-identical devices? (2) How do the platform architecture and workload characteristics influence fingerprint quality?

We employ a dual evaluation approach. First, we apply supervised classification using six machine learning algorithms to measure closed-set identification performance, providing practical recognition accuracy metrics. Second, we conduct centroid-based distance analysis to quantify feature space geometry independent of classifier architecture. For each workload–platform combination, device centroids are computed as the mean feature vector across all windowed samples collected during the session, enabling distance-based separation metrics that reveal the intrinsic separability of manufacturing variations. This dual approach allows both performance assessment and interpretability of fingerprint discriminability.

#### 3.3.1. Classification Performance Metrics

We trained six supervised classifiers spanning diverse algorithmic families: Random Forest (RFC), Support Vector Machine (SVM), K-Nearest Neighbors (K-NN), Logistic Regression (LR), Gaussian Naive Bayes (GNB), and Decision Tree (DT). All classifiers were evaluated via five-fold stratified cross-validation with Min-Max normalization. Performance is quantified through accuracy, precision, recall, F1-score, and confusion matrices.

#### 3.3.2. Distance-Based Separation Metrics

Device separation quality is further assessed via two quantitative metrics that complement classification accuracy by measuring geometric separability in feature space.

*(a) d-prime* measures the standardized distance between intra-device and inter-device distance distributions:

(2) $$d^{\prime }=\frac{|\mu_{\mathrm{inter}}-\mu_{\mathrm{intra}}|}{\sqrt{0.5(\sigma_{\mathrm{inter}}^{2}+\sigma_{\mathrm{intra}}^{2})}}$$

where $\mu_{\mathrm{inter}}$ and $\mu_{\mathrm{intra}}$ represent mean inter-device and intra-device distances, respectively, and $\sigma_{\mathrm{inter}}^{2}$ and $\sigma_{\mathrm{intra}}^{2}$ denote their corresponding variances. Following signal detection theory conventions [38], $d^{\prime }>1.5$ indicates strong separation, 1.0–1.5 suggests moderate separation, and <1.0 indicates weak separation.

*(b) Separation ratio* computes the ratio of inter- to intra-device mean distances:

(3) $$\mathrm{separation}\;\mathrm{ratio}=\frac{\mu_{\mathrm{inter}}}{\mu_{\mathrm{intra}}}$$

Higher ratios reflect stronger device discriminability, with values exceeding 2.0 indicating inter-device distances at least twice intra-device distances.

#### 3.3.3. Cross-Platform Comparison and Architecture Impact Analysis

We evaluate seven ESP32-WROOM boards (Espressif Systems, Shanghai, China; 32-bit dual-core, 240 MHz) and seven Arduino Uno R3 boards (Arduino S.r.l., Monza, Italy; eight-bit single-core, 16 MHz) under identical workloads. Both platforms lack hardware security primitives, making them representative of vulnerable low-cost IoT devices. Architectural details and platform rationale are provided in Section 4.

#### 3.3.4. Workload Impact Analysis

Fingerprints are also evaluated across multiple workloads within each platform to identify which computational patterns most effectively expose manufacturing variations. Workload specifications are provided in Section 4.3. Our hypothesis is that tasks stressing different subsystems (compute, memory, communication) reveal distinct manufacturing variations, though more complex workloads could also produce averaging effects that obscure fine-grained differences.

### 3.4. Multi-Run Temporal Analysis

We conduct a multi-run evaluation to assess fingerprint temporal stability across four measurement sessions performed on separate days spanning a 45-day period. All devices undergo power tracing at each session, and features are extracted identically to the single-run pipeline. Segmentation yielded different numbers of complete execution windows per session: Run 1 (71 windows × 1152 samples), Run 2 (62 × 1153), Run 3 (117 × 1113) and Run 4 (19 × 1115), where the second figure is the number of samples retained per window after length uniformization. These counts differ because each session was a fixed 45-min acquisition rather than a fixed number of cycles, so the number of complete sensor-read-and-transmit cycles captured depends on how network association and transmission timing fell within that window; sessions in which the node spent longer in connection or retransmission yield fewer complete cycles. To make sessions directly comparable, the cross-session alignment step of Section 4.4 truncates every device and every session to the smallest common window count, which is the 19 windows of Run 4. The dataset entering all multi-session analyses is therefore exactly 7 devices × 4 sessions × 19 windows = 532 feature vectors of 17 dimensions each. We report this explicitly because it, rather than the raw per-session counts, is the sample size on which every multi-session result in Section 5 rests.

Our analysis examines (1) per-session separation quality (each day analyzed independently), (2) cross-session degradation (training/testing across multiple sessions), and (3) embedding-based robustness (neural representations vs. statistical features).

#### 3.4.1. Per-Session Analysis

For each measurement day, we repeat the complete single-run evaluation independently. We use the same metrics—d-prime, separation ratio, and classification accuracy—to evaluate the fingerprint quality. Results reveal whether device separation remains strong at each time point when all training and testing data come from the same day.

#### 3.4.2. Cross-Session Degradation

The real challenge in device fingerprinting emerges when recognition must span different time periods. To assess whether fingerprints remain stable across temporal gaps, we implement a cross-session evaluation protocol: training on data from earlier sessions and testing on data from later sessions. This design directly probes whether fingerprints degrade over time and whether feature representations differ in their temporal robustness.

As discussed in Section 5.2, this temporal analysis reveals significant differences between statistical features and learned representations. These findings motivated an additional investigation into more robust feature representations, detailed in Section 3.4.3.

#### 3.4.3. Neural Embedding Framework

To address this temporal instability, we adopt a neural network approach designed to learn more robust feature representations that can better handle temporal variations. Our neural network uses a ResNet-inspired design [39] that balances learning capacity with computational efficiency suitable for IoT devices. Its compact 64-dimensional output keeps the approach practical for deployment on resource-constrained devices while retaining the information the fingerprint depends on.

We train the network using a combined loss function with two objectives. Triplet loss [40] encourages intra-device compactness by pushing samples from the same device closer together while pulling samples from different devices apart. Additive margin Softmax (AM-Softmax) [41] enforces angular separation between device classes, creating more distinct decision boundaries.

The encoder maps the 17-dimensional feature vector through fully connected layers of 256 and 128 units to a 64-dimensional L2 normalized embedding, with ReLU activations, dropout of 0.35 after the first layer, batch normalization on the second, and a learned linear projection forming the residual shortcut from the 256-unit layer to the 128-unit layer. Excluding the AM-Softmax head, this is 78,912 parameters; the head adds 64n for *n* enrolled devices, giving 79,360 at n=7. Training minimizes an equally weighted sum (λ=0.5 each) of batch-hard triplet loss with margin 1.2 and AM-Softmax cross-entropy with scale 30 and margin 0.35, using AdamW at a learning rate of $1\times 10^{-4}$ with weight decay $1\times 10^{-4}$ for a fixed 80 epochs with no early stopping. Batches are formed by *P*–*K* sampling with P=6 devices and K=8 windows per device, giving a batch size of 48; Gaussian jitter with σ=0.02 is added to each sample as augmentation. Verification uses cosine similarity to the enrolled centroid. All random number generators (Python 3.13.15, NumPy 2.1.3 and PyTorch 2.11.0+cu128, including CUDA) are seeded with 42, and cuDNN is placed in deterministic mode; the same seed is used for the preprocessing pipeline and for all bootstrap resampling. The relative contribution of each architectural and training component is quantified through ablation analysis in Section 5.5.

At evaluation time, device identity verification is performed without a separate classifier. For each enrolled device, a reference centroid is computed as the L2-normalized mean of its training embeddings. Test samples are scored by cosine similarity to each device’s centroid, and scores are z-normalized using impostor statistics from the validation set. A sample is accepted as belonging to a device if its normalized score exceeds a calibrated threshold; otherwise, it is rejected. This same scoring mechanism is applied identically to both raw statistical features and neural embeddings, ensuring that observed performance differences reflect the feature representation rather than the decision mechanism.

We used three evaluation protocols to assess how well neural embeddings maintain their robustness over time vs. statistical features. Each tests a different aspect of temporal generalization.

*a. Temporal-mixed split:* All samples from the four measurement sessions are randomly pooled and split into 60% training, 20% validation, and 20% testing. This protocol evaluates whether the model can learn reliable device fingerprints when training data spans the full 45-day period.

*b. Five-fold cross-validation:* We partition the data into five folds. For each fold, four folds (80%) are used for training, while the held-out fold is split equally between validation and testing. This protocol estimates robustness by averaging performance across different temporal partitions of the data.

*c. Leave-One-Run-Out (LORO):* Applied to the Networked Sensor Monitoring sessions, each of the four measurement sessions is held out in turn. For a given device and a given held-out session, the encoder and the device centroid are built from that device’s remaining three sessions (3×19=57 windows), and the held-out session’s 19 windows are split by position into the first nine, used for threshold calibration, and the remaining 10, used for testing. Rotating the held-out session over all four gives four folds per device and 7×4=28 device-fold results, which is the sample size reported for the LORO protocol in Section 5.3. No window from a held-out session contributes to training, to centroid construction, or to threshold calibration for any other fold. For comparison, the temporal-mixed protocol pools each device’s 76 windows and splits them 60/20/20 (45 train, 15 validation, 16 test), and the five-fold cross-validation protocol yields 60 training windows with the held-out 16 split into roughly eight validations and eight tests, giving 7×1=7 and 7×5=35 device-fold results respectively.

The three protocols differ in how much temporal separation they enforce, and we read them in that order. The temporal-mixed and cross-validation protocols draw training and test windows from the pooled set of all four sessions and so characterize recognition when enrollment data spans the full observation period. The LORO protocol additionally requires that no window from the evaluated session contribute to training, to centroid construction or to threshold calibration, which is the condition a deployed verifier meets when it is asked to recognize a device on a day it has not seen. Read together, they show whether neural embeddings hold device identity across the 45-day period better than statistical features and whether temporal diversity in the training data improves generalization to later sessions. All headline results reported in this work—the 93.9% true positive rate at 9.9% false acceptance, the comparison against statistical features in Section 5.3, and the ablation study of Section 5.5—are obtained under LORO protocol.

Across all three protocols, the same fitting discipline applies. Within every fold, the min–max scaler is fitted on the device’s training partition and applied unchanged to its validation and test partitions, and the reference centroid is computed from training embeddings alone, so no test statistic enters any transformed value or any enrolled reference. Feature screening (Section 3.2) is carried out once over the pooled dataset and uses no device labels: its criteria are sample count, cardinality and variance, so the retained feature set is common to every device and independent of how the data is subsequently partitioned—a precondition for the cross-device distance comparisons of Section 5, since a feature set chosen per device would leave those distances measured on different scales and so not comparable.

#### 3.4.4. Open-Set Evaluation Protocol

Intrusion detection requires distinguishing between known (enrolled) devices and unknown devices (impostors or substitutes). This necessitates an open-set detection system, where the system must recognize known devices while rejecting unknowns, in contrast to closed-set classification, which assumes all test samples belong to one of the known classes.

**Figure 3:**
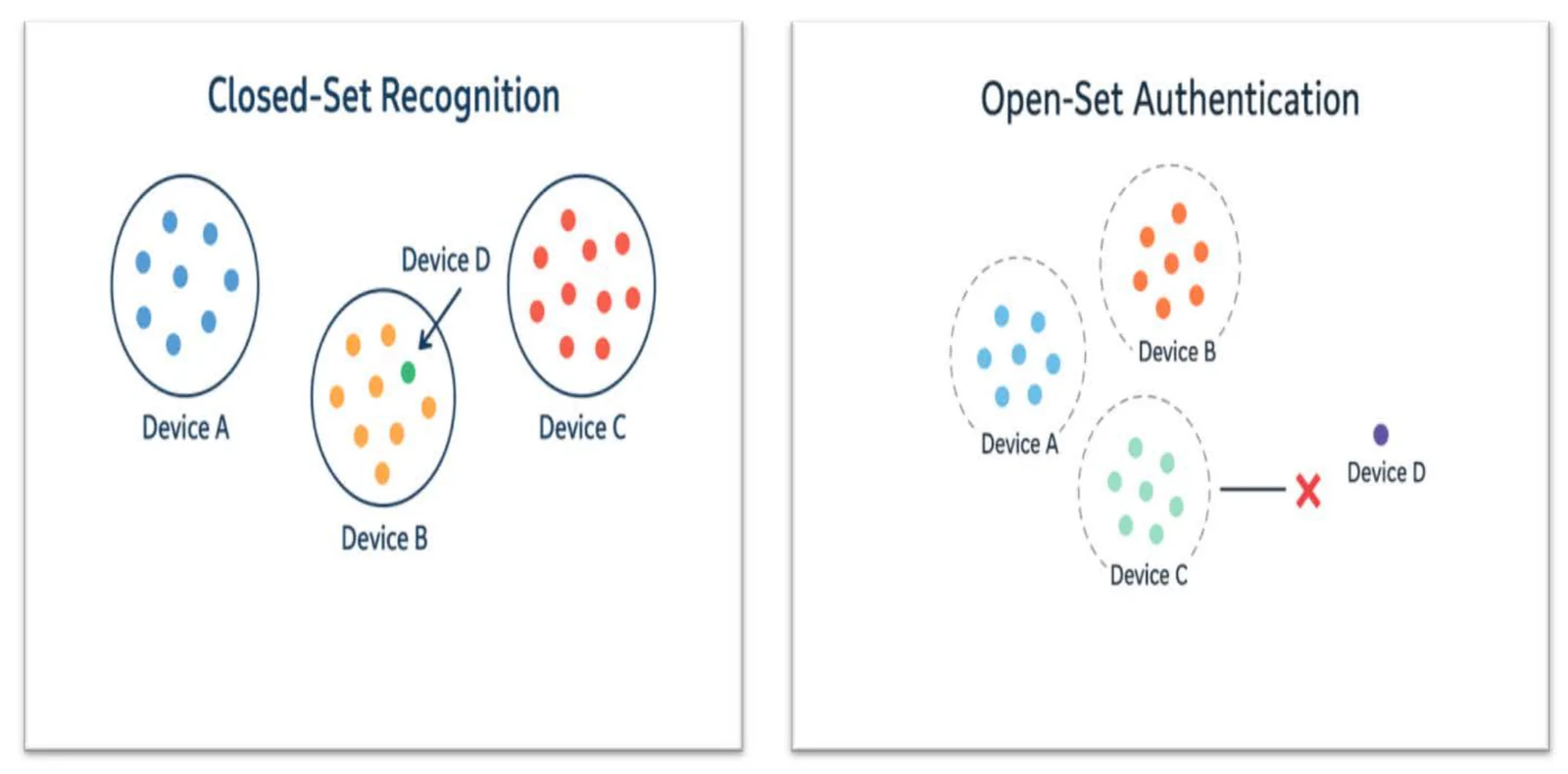
Closed-set vs. open-set decision concepts. (**Left**) Closed-set classification assigns every probe to one of the enrolled device classes, even if the probe is from an unknown device (Device D is incorrectly assigned to Device B’s cluster). (**Right**) Open-set authentication rejects probes that fall outside calibrated acceptance boundaries (dashed circles), correctly identifying Device D as an intruder.

Closed-set classification forces unknown devices to be misclassified as known ones. Open-set authentication addresses this using centroid-based cosine similarity scoring, as described in Section 3.4.3. A sample is accepted if its similarity score exceeds a calibrated threshold; otherwise, it is rejected. Figure 3 illustrates this fundamental difference.

Open-set recognition is evaluated within the LORO protocol described above, following the open-set authorization paradigm of [42] in which enrolled devices must be accepted while unauthorized devices are rejected. In each held-out session, every enrolled device is treated in turn as the verification target. The remaining devices of the batch supply impostor queries drawn from that same session, so no impostor window has been seen during training, and rejection is measured against physically distinct units of the same model under temporal shift. Section 5 reports a second condition in which the impostor identity is withheld from training in full.

Three threshold strategies are evaluated to illustrate the rejection-acceptance tradeoff, following standard biometric evaluation practice [43]. The **FRR-optimized threshold** (fifth percentile of genuine scores) minimizes the False Rejection Rate (FRR) and therefore prioritizes usability by accepting most legitimate devices. The **FAR-optimized threshold** (95th percentile of impostor scores) minimizes the False Acceptance Rate (FAR) and therefore prioritizes security by rejecting most impostors. The **EER threshold** is the operating point at which FRR and FAR are equal. We report it as a single-number balanced reference that allows comparison across representations and protocols under equal weighting of the two error types; it is not optimal in general, since a deployment should select its operating point according to application-specific costs of false acceptance and false rejection. All thresholds are calibrated on validation-set score distributions and applied without modification to test data, ensuring no information leakage from the evaluation set into threshold selection. Impostor scores entering calibration are drawn from the validation partitions of the remaining devices and normalized per fold before the threshold is set; the partition sizes for each protocol are given in Section 3.4.3.

**Figure 4:**
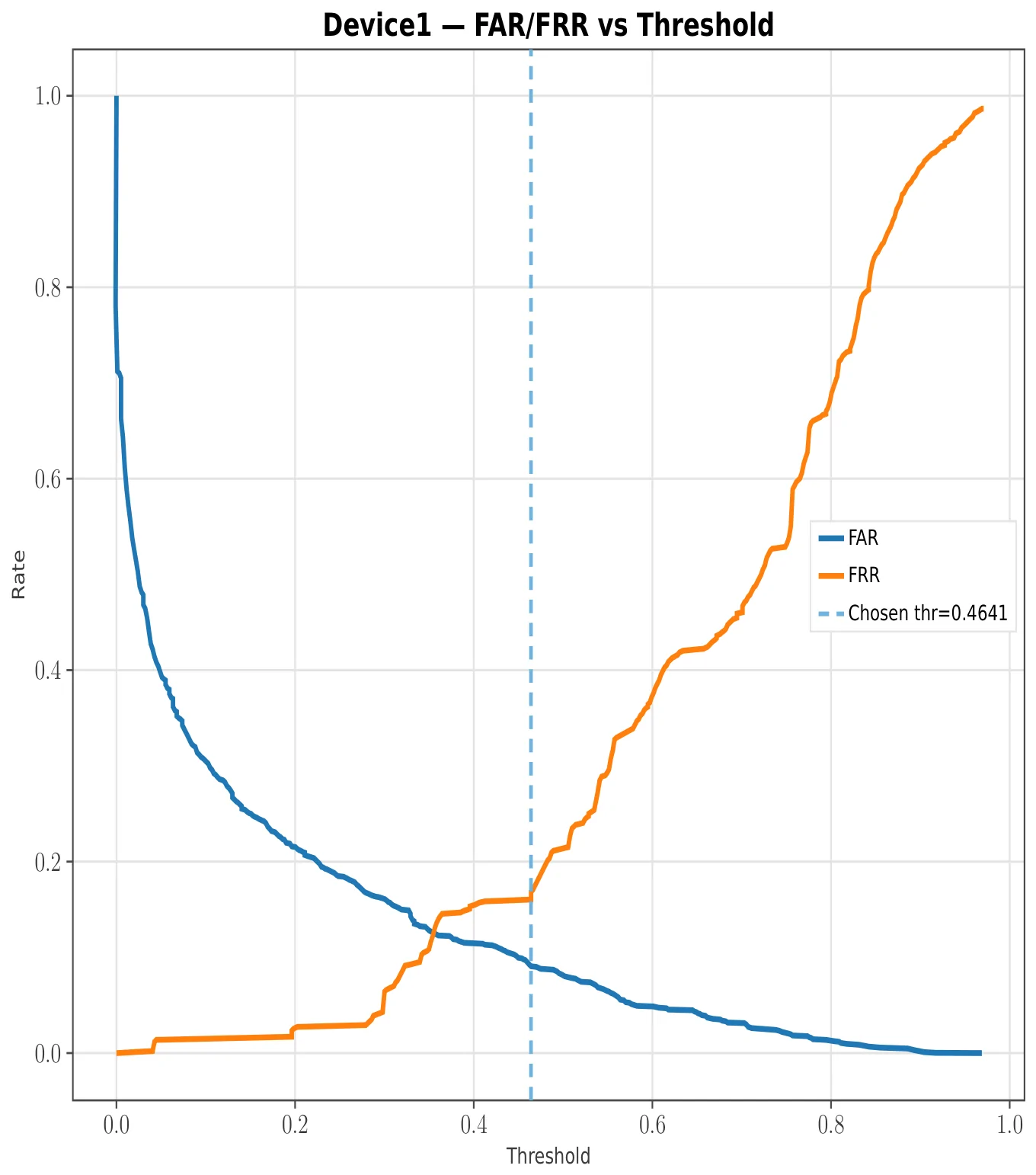
False acceptance and false rejection rates as a function of the acceptance threshold, shown for one enrolled device. As the threshold rises, the false acceptance rate falls and the false rejection rate grows; the equal error rate is the threshold at which the two curves meet. The dashed line marks the operating point selected for this device. The three threshold strategies correspond to different positions along these curves.

Figure 4 visualizes this trade-off for a representative device: the FRR-optimized and FAR-optimized thresholds lie toward opposite ends of the curves, and the EER threshold at their intersection. Results for each strategy are reported separately.

Beyond testing against simple unknown devices, we evaluate robustness under three impostor scenarios, all defined in the learned embedding space. Let $e_{\mathrm{imp}}$ denote the embedding of a non-enrolled device and $\mu_{t}$ the centroid of the target device under test. The first scenario uses unmodified impostor embeddings,

(4) $$z_{\mathrm{real}}=e_{\mathrm{imp}},$$

and yields the Real FAR (${\mathrm{FAR}}_{\mathrm{real}}$), the most realistic case, in which genuine non-enrolled devices from the same manufacturing batch are presented to the verifier. The second scenario models a quantization-and-noise perturbation representing a reduced-fidelity duplicate,

(5) $$z_{\mathrm{hw}}=Q_{8}\left(e_{\mathrm{imp}}\right)+\eta ,\;\eta ∼N\left(0,\sigma^{2}I\right),\;\sigma =0.02,$$

where $Q_{8}\left(\cdot \right)$ is uniform eight-bit quantization, yielding the Hardware FAR (${\mathrm{FAR}}_{\mathrm{hw}}$). The third scenario, the adversary scenario, shifts an impostor embedding a fixed fraction toward the target centroid,

(6) $$z_{\mathrm{adv}}=\alpha \;e_{\mathrm{imp}}+\left(1-\alpha \right)\;\mu_{t},\;\alpha =0.85,$$

yielding the Adversary FAR (${\mathrm{FAR}}_{\mathrm{adv}}$). This scenario is a sensitivity analysis in the representation space rather than a model of a physically realized attack: α parameterizes how far an impostor embedding is displaced toward the target. We set α=0.85 so that the impostor retains most of its own signature while acquiring a 15% component of the target, which probes the acceptance boundary without collapsing the two identities into one; smaller α raises false acceptance and larger α lowers it. Relating a value of α to a specific physical cloning capability would require measurements from deliberately manufactured near-clones, which we identify as future work. We emphasize that all three perturbations are applied to embeddings rather than to raw power traces, and thus characterize behavior in the learned representation space rather than reproducing a specific physical attack.

We quantify open-set detection effectiveness through the True Positive Rate (TPR), False Acceptance Rate (FAR) reported separately for each attack scenario (Real FAR, Adversary FAR, Hardware FAR), and Equal Error Rate (EER), which identifies the threshold where false rejections equal false acceptances.

### 3.5. Statistical Validation Methodology

To ensure our findings meet rigorous statistical standards, we supplement performance metrics with effect size analysis [44], power calculations, and uncertainty quantification. These statistical measures help determine whether observed differences are not only statistically significant but also practically meaningful.

#### 3.5.1. Effect Size Analysis

Raw performance differences can be misleading without context about their magnitude. We compute Cohen’s *d* [45] to measure the standardized difference between group means:

(7) $$d=\frac{|\mu_{1}-\mu_{2}|}{\sigma_{\mathrm{pooled}}},\;\sigma_{\mathrm{pooled}}=\sqrt{\frac{\left(n_{1}-1\right)s_{1}^{2}+\left(n_{2}-1\right)s_{2}^{2}}{n_{1}+n_{2}-2}}$$

where *d* = Cohen’s *d* (effect size), $\mu_{1}$ = mean of group 1, $\mu_{2}$ = mean of group 2, $\sigma_{\mathrm{pooled}}$ = pooled standard deviation, $n_{1}$ = sample size of group 1, $n_{2}$ = sample size of group 2, $s_{1}^{2}$ = variance of group 1, $s_{2}^{2}$ = variance of group 2.

We calculate effect sizes for two critical comparisons: first, intra-device versus inter-device distances to quantify separation quality, and second, statistical features versus neural embeddings to assess temporal robustness improvements. Following established conventions, we interpret d≥0.8 as large effects, 0.5–0.79 as medium, 0.2–0.49 as small, and d<0.2 as negligible [46].

#### 3.5.2. Statistical Power Assessment

We conduct post hoc power analysis to calculate achieved statistical power (1−β), where β is the Type II error rate (the probability of failing to detect an effect that is genuinely present). The non-centrality parameter is calculated as

(8) $$\lambda =d\times \sqrt{\frac{n_{1}n_{2}}{n_{1}+n_{2}}}$$

where *d* is Cohen’s *d* and $n_{1},n_{2}$ are the sample sizes of the two groups.

Power is then computed using a non-central t-distribution with degrees of freedom (df) = $n_{1}+n_{2}-2$. Power exceeding 80% indicates adequate sample size for reliable conclusions, 60–80% suggests moderate validity, and values below 60% warrant cautious interpretation of results [47].

#### 3.5.3. Sample Size Planning

To evaluate the scalability of our framework, we perform a prospective power analysis to determine the sample sizes required for detecting small (d=0.2), medium (d=0.5), and large (d=0.8) effects [45]. Sample size is calculated using the formula:

(9) $$n=2{\left[\frac{z_{\alpha }+z_{\beta }}{d}\right]}^{2}$$

where α = Type I error rate, β = Type II error rate, $z_{\alpha }$ = translates α=0.05 into a z-score (1.96), and $z_{\beta }$ = translates β=0.20 into a z-score (0.84).

These calculations estimate the sample sizes needed to reliably detect effect sizes of varying magnitudes when extending the framework to larger device populations.

#### 3.5.4. Uncertainty Quantification

For open-set framework metrics (TPR, FAR, EER), we compute 95% confidence intervals [48] using bootstrap resampling with 1000 iterations. This non-parametric approach provides robust uncertainty estimates without requiring assumptions about underlying distributions, enabling reliable interpretation of detection performance variability.

Confidence intervals were computed for all evaluation protocols (fixed split, cross-validation, and LORO cross-session) and thresholding strategies (FRR-optimized, FAR-optimized, and EER). For clarity, representative results and detailed comparisons are discussed in Section 5.

## 4. Experimental Setup

This section details the experimental methodology for power side-channel fingerprinting of batch-identical commercial microcontrollers. We describe the hardware configuration, measurement setup, workloads, data structure, and evaluation timeline. The focus is on reproducibility and systematic evaluation across multiple temporal sessions.

### 4.1. Hardware and Device Pool

The device pool consists of seven ESP32-WROOM-32 (240 MHz, 32-bit dual-core Xtensa LX6) development boards and seven Arduino Uno R3 (16 MHz, 8-bit AVR ATmega328P) boards. Each family is sourced from a single manufacturing batch to ensure that observed inter-device differences reflect manufacturing variations rather than batch-to-batch process shifts.

These platforms span the architectural extremes of the low-cost COTS microcontroller space. Arduino Uno is 8-bit CISC, single-core, with no cache or wireless subsystem. ESP32 is 32-bit RISC, dual-core, with pipelined execution, multi-level caching, and integrated Wi-Fi/Bluetooth. Both operate in the milliwatt range, where manufacturing-induced differences are smaller than those reported for higher-power platforms [11,12,17]. By testing at these two ends of the spectrum, we obtain initial evidence that fingerprinting is viable across different hardware complexities rather than being an artifact of a single architecture.

### 4.2. Measurement Setup

**Figure 5:**
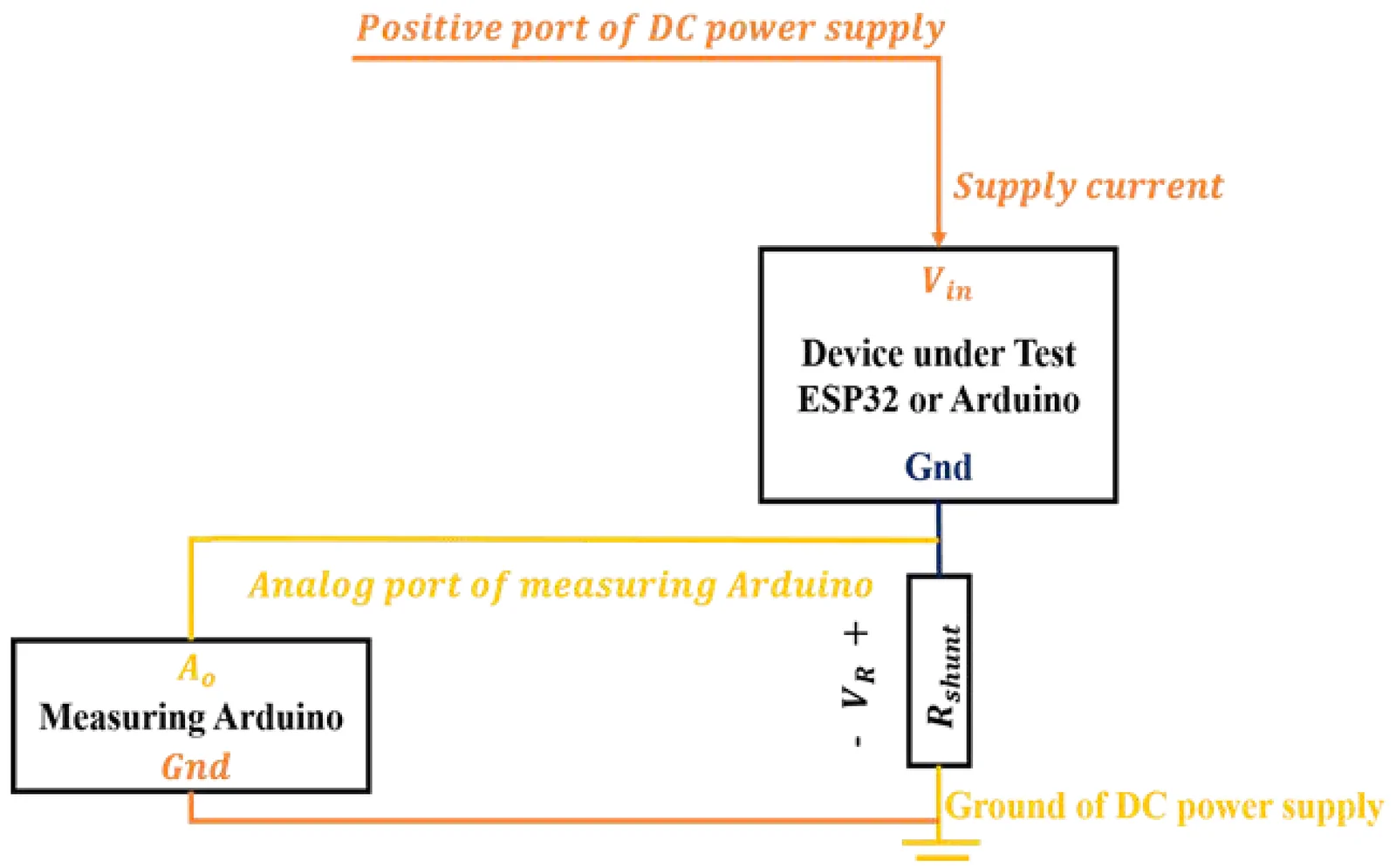
Power measurement setup showing shunt resistor placement in the common ground path. Voltage drop across the 4.5 Ω precision resistor is amplified and digitized to capture device-specific power consumption signatures.

Device power draw is observed non-intrusively through a precision shunt resistor inserted in the common ground path between the power supply and the device under test, as shown in Figure 5.

#### 4.2.1. Data Acquisition and Experimental Consistency

A single dedicated Arduino Uno serves as the measurement unit for all devices across all experimental sessions, ensuring consistent measurement infrastructure. Its 10-bit ADC samples the voltage drop across the shunt resistor at rates between 100 Hz and 1 kHz, depending on workload requirements. All raw traces are stored as CSV files with device, workload, and timestamp metadata for full traceability. Raw samples undergo offline preprocessing according to Section 3.2. Any minor environmental variations in the measurement unit (temperature, supply voltage) affect all devices equally and are orthogonal to the manufacturing-induced variations under investigation.

Environmental conditions were not recorded during sessions; assumptions and implications are discussed in Section 3 and Section 6, respectively.

#### 4.2.2. Measurement Principles

The signal digitized by the acquisition unit is the voltage $v_{\mathrm{sh}}\left(t\right)$ developed across the shunt sensing resistor $R_{\mathrm{sh}}$. The supply is held at $V_{\mathrm{dd}}=5$ V and $R_{\mathrm{sh}}$ is a 4.5 Ω precision resistor, so the recorded shunt voltage $v_{\mathrm{sh}}\left(t\right)$, the supply current i(t), and the power drawn from the supply p(t) satisfy

(10) $$i\left(t\right)=\frac{v_{\mathrm{sh}}\left(t\right)}{R_{\mathrm{sh}}},\;p\left(t\right)=V_{\mathrm{dd}}\;i\left(t\right)=\frac{V_{\mathrm{dd}}}{R_{\mathrm{sh}}}\;v_{\mathrm{sh}}\left(t\right).$$

Each of the three electrical quantities is therefore a fixed multiple of the others, and we report the signal in the units in which it is acquired. This choice does not affect any result. Of the descriptors defined in Section 3.2, the location and dispersion statistics scale linearly with the signal, while skewness, spectral entropy and the peak count are scale-invariant, and the per-device min–max normalization applied before analysis removes any common multiplicative factor exactly. Every separation ratio, $d^{\prime }$ value, and detection rate reported in this work is consequently identical whether the traces are expressed in volts, amperes or watts.

A single sensing resistor serves every device in every session, so its value is a common factor across the entire dataset. Differences between devices, which are what device discrimination measures, are therefore independent of its absolute value, and any session-to-session variation in the measurement chain enters the evaluation directly, since verification is tested on sessions held out from training (Section 5.2). Following established usage in this literature [10,11,21], we describe the modality as power side-channel fingerprinting throughout.

### 4.3. Workloads

Workloads were designed to stress different microcontroller subsystems—computational logic, memory access patterns, and I/O peripherals—while maintaining deterministic execution patterns. Table 2 summarizes the three workloads, the hardware components each targets, and its role in the evaluation. Each workload executes identically across all devices within a family, ensuring consistent power consumption measurements and enabling direct cross-device comparison.

The Compute–Hash–Sleep Loop is used on both ESP32 and Arduino Uno platforms for baseline cross-platform comparison, enabling assessment of how architectural differences affect fingerprinting effectiveness. For same-platform multi-workload evaluation, ESP32 boards additionally execute the Networked Sensor Monitoring Task, while Arduino Uno boards additionally execute the Bubble Sort Algorithm, enabling platform-specific workload analysis.

The Networked Sensor Monitoring Task on ESP32 also serves as the workload for multi-run temporal evaluation, providing a 45-day assessment of fingerprint stability under realistic operational conditions.

### 4.4. Data Structure and Windowing

**Figure 6:**
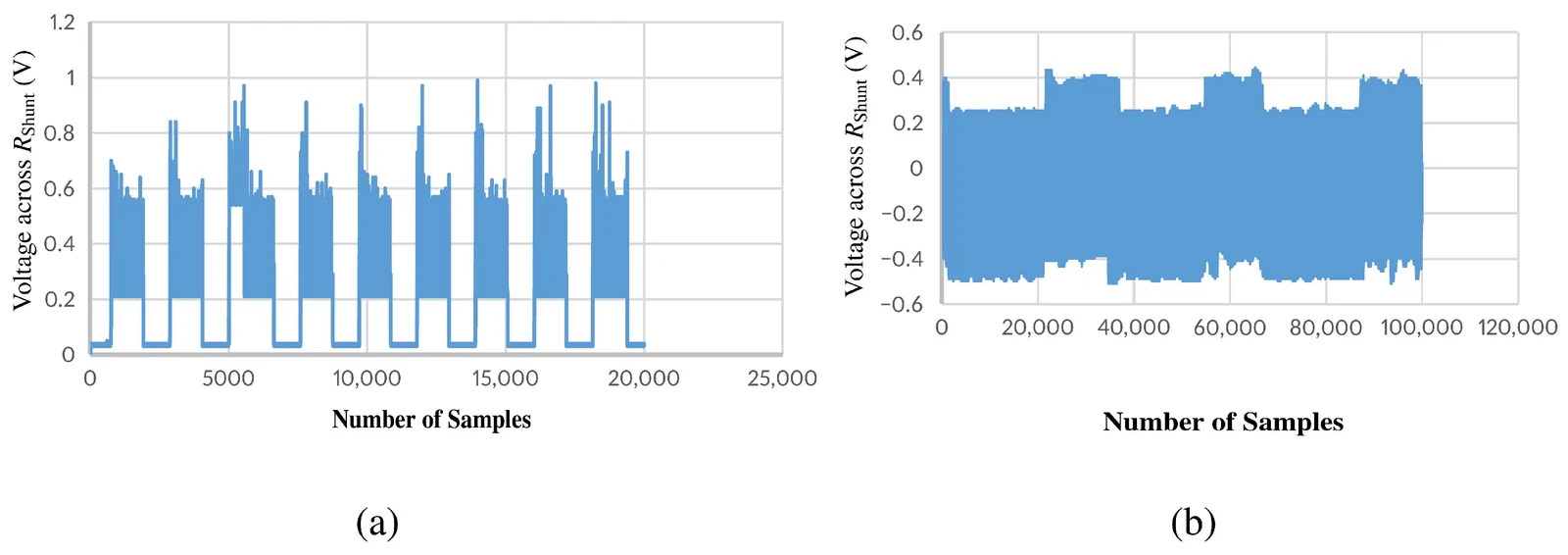
Power consumption patterns during task execution. (**a**) Shunt voltage profiling of ESP32 nodes showing repetitive execution cycles with active and sleep segments. (**b**) Shunt voltage profiling of Arduino Uno boards demonstrating task execution and idle for window extraction.

Power traces are segmented into fixed-length windows corresponding to complete task execution cycles. Figure 6 illustrates the repetitive patterns in power consumption profiles during task execution. Windowing extracts task execution intervals from continuous power measurements, creating uniform temporal boundaries for consistent feature extraction.

Each window is normalized for length and sample count, ensuring uniform feature extraction across devices and runs. The resulting data structure is organized as Device × Run × Window × Feature Array, supporting comprehensive temporal analysis and statistical evaluation.

#### Window Normalization Protocol

Although all boards execute identical tasks under similar conditions, the captured datasets still show natural variations in window count and execution timing. To ensure consistency, a systematic normalization protocol is implemented:

**(a) Window Extraction and Segmentation:** Window boundaries are identified through zero-crossing detection in the shunt voltage traces, with workload-specific preprocessing. After edge detection, length constraints filter spurious transients.

For the Compute–Hash–Sleep workload, the power traces naturally contain zero-valued idle intervals between execution cycles due to the deep-sleep phase, so no voltage thresholding is required; the segmentation algorithm directly detects rising-edge (zero to non-zero) and falling-edge (non-zero to zero) transitions in the raw measurements. Only windows containing 30–39 samples (ESP32) or 35–44 samples (Arduino) are retained, corresponding to complete single-cycle execution intervals. These narrow ranges reflect the short, deterministic execution cycles of the hash-sleep workload, which completes in tens of milliseconds.

For the Networked Sensor Monitoring workload, which lacks a full power-down phase, a voltage threshold of 0.05 V is applied to distinguish active execution intervals from low-power idle periods: values at or below this threshold are set to zero before edge detection. This value was determined empirically by inspecting raw shunt voltage traces across all devices and sessions, where it consistently separates quiescent baseline current from active task execution. Windows shorter than 500 samples are discarded to ensure each window captures a complete sensor-read and network transmission cycle.

**(b) Sample Count Uniformization:** Window lengths are standardized through truncation. Within each device, all windows are trimmed to match the shortest window length, retaining only the final samples to preserve the characteristic active computation phase while discarding initial transient behavior.

**(c) Window Count Standardization:** The minimum number of windows captured across all devices determines the final dataset size. All devices are then truncated to this minimum window count, ensuring equal representation across the device pool.

**(d) Multi-Run Consistency:** Across all four temporal sessions, window extraction thresholds and normalization parameters remain fixed. Feature matrices from different sessions are aligned to identical dimensions using row-matching procedures, enabling valid temporal comparisons while preserving underlying manufacturing-induced power signature characteristics.

This ensures statistical validity while preserving underlying power signature characteristics.

### 4.5. Timeline and Experimental Protocol

The experimental evaluation uses a structured timeline to assess device performance. It targets both baseline device separability and temporal stability across realistic IoT deployment periods.

#### 4.5.1. Single-Run Evaluation (Day 0)

All devices are evaluated to establish baseline fingerprint separability using the Compute–Hash–Sleep workload on both platforms (Section 5.1.1).

**Measurement Protocol:** Within each session, all devices execute the same workload consecutively. Each device is measured individually in sequence with unchanged infrastructure, ensuring differences reflect manufacturing variations.

Workload-dependent effects are examined within each platform (Section 5.1.3 and Section 5.1.2).

#### 4.5.2. Multi-Run Temporal Analysis (ESP32 Only)

ESP32 devices execute the Networked Sensor Monitoring Task across four measurement sessions spanning 45 days. The initial session (Day 0) serves as enrollment, followed by a second session roughly one month later. The third and fourth sessions are conducted one and two weeks after the second session, respectively. This spacing assesses short-term fingerprint stability under laboratory conditions. Each evaluation consists of 45 min of continuous data capture per device per workload to ensure sufficient sample collection for reliable feature extraction and statistical analysis.

## 5. Results

This section presents evaluation results from our single-run baseline analysis, multi-run temporal assessment, neural embedding findings, and statistical power validation.

### 5.1. Single-Run Baseline Analysis

#### 5.1.1. Architectural Impact on Fingerprinting

On Day 0, we evaluated how well manufacturing variations could distinguish between same-batch devices for each platform. ESP32 and Arduino Uno families executed identical Compute–Hash–Sleep workloads. Table 3 presents baseline separation metrics using distance-based measures.

ESP32 showed greater manufacturing variation signal than Arduino Uno, with a 1.80× higher separation ratio (3.06 vs. 1.70) and 2.03× larger d-prime ($d^{\prime }$ = 2.54 vs. 1.25). Intra-device distance was 26% tighter (0.323 vs. 0.437), while inter-device distance was 33% larger (0.988 vs. 0.740). Six supervised learning algorithms achieved 84.9–96.1% F1-scores on ESP32 compared to 64.7–71.1% on Arduino, with Random Forest performing best on both platforms (ESP32: 96.1%, Arduino: 71.1%).

**Figure 7:**
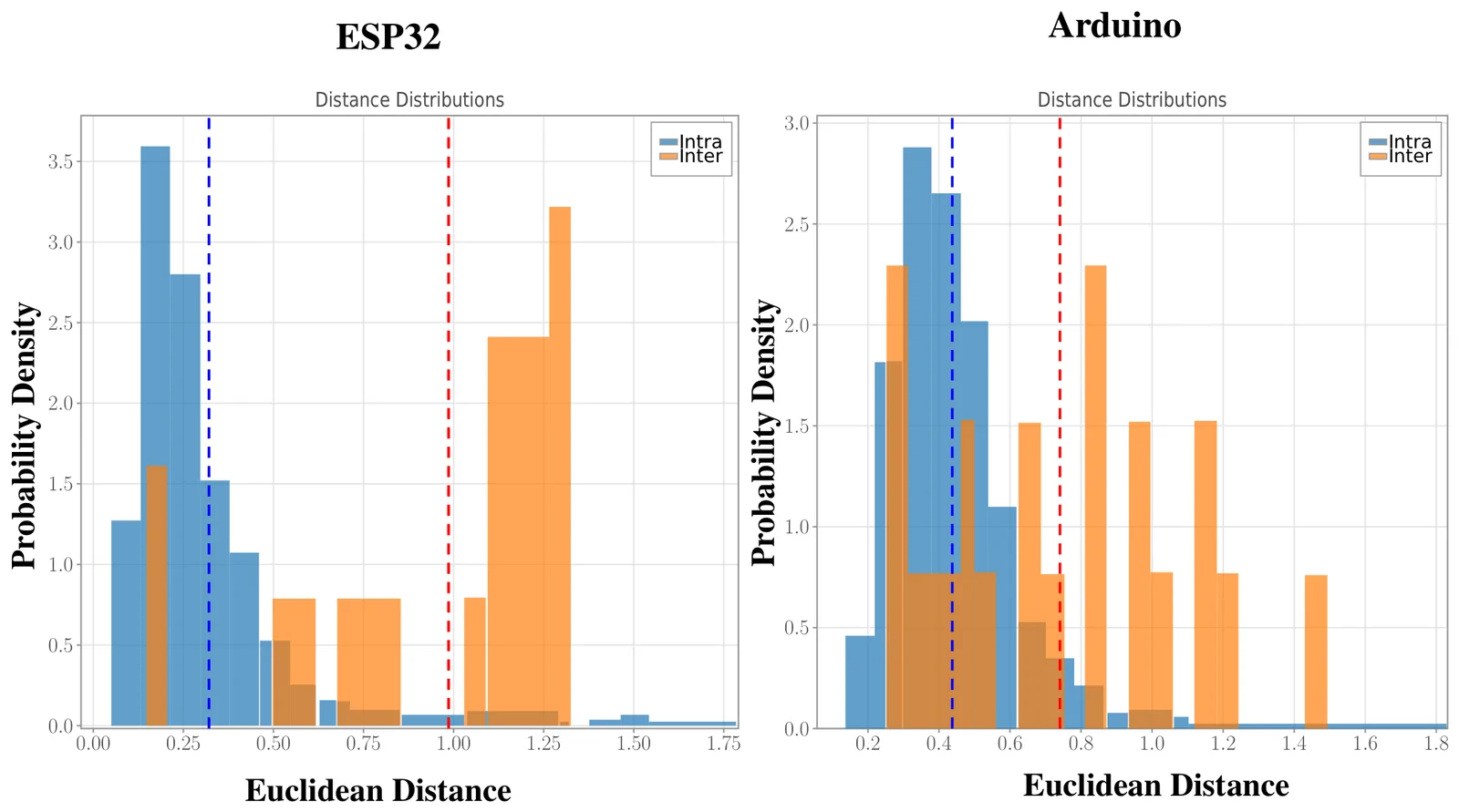
Distance Distribution Comparison between ESP32 and Arduino Platforms.

Figure 7 visualizes the probability density distributions of Euclidean distances for both platforms. The blue histogram represents intra-device distances (variations within the same device), with the blue dashed line indicating the intra-class mean. The orange histogram represents inter-device distances (variations between different devices), with the red dashed line indicating the inter-class mean.

ESP32 demonstrates superior separability as evidenced by the minimal overlap between its intra- and inter-device distributions compared to the significant overlap observed in the Arduino plot under the same workload and measurement chain. The two families are compared here as whole systems: they differ concurrently in architecture, process node, on-board regulation, board layout, clock rate, workload timing and absolute current magnitude, so the comparison establishes which platform yields the stronger fingerprint rather than which of those properties produces it. Resolving the latter would call for a series of platforms varying one property at a time—for instance, families sharing a process node but differing in core count, or a single die measured at several clock rates—which we identify as a productive direction for extending this analysis.

#### 5.1.2. Workload Impact on Arduino Platform

**Figure 8:**
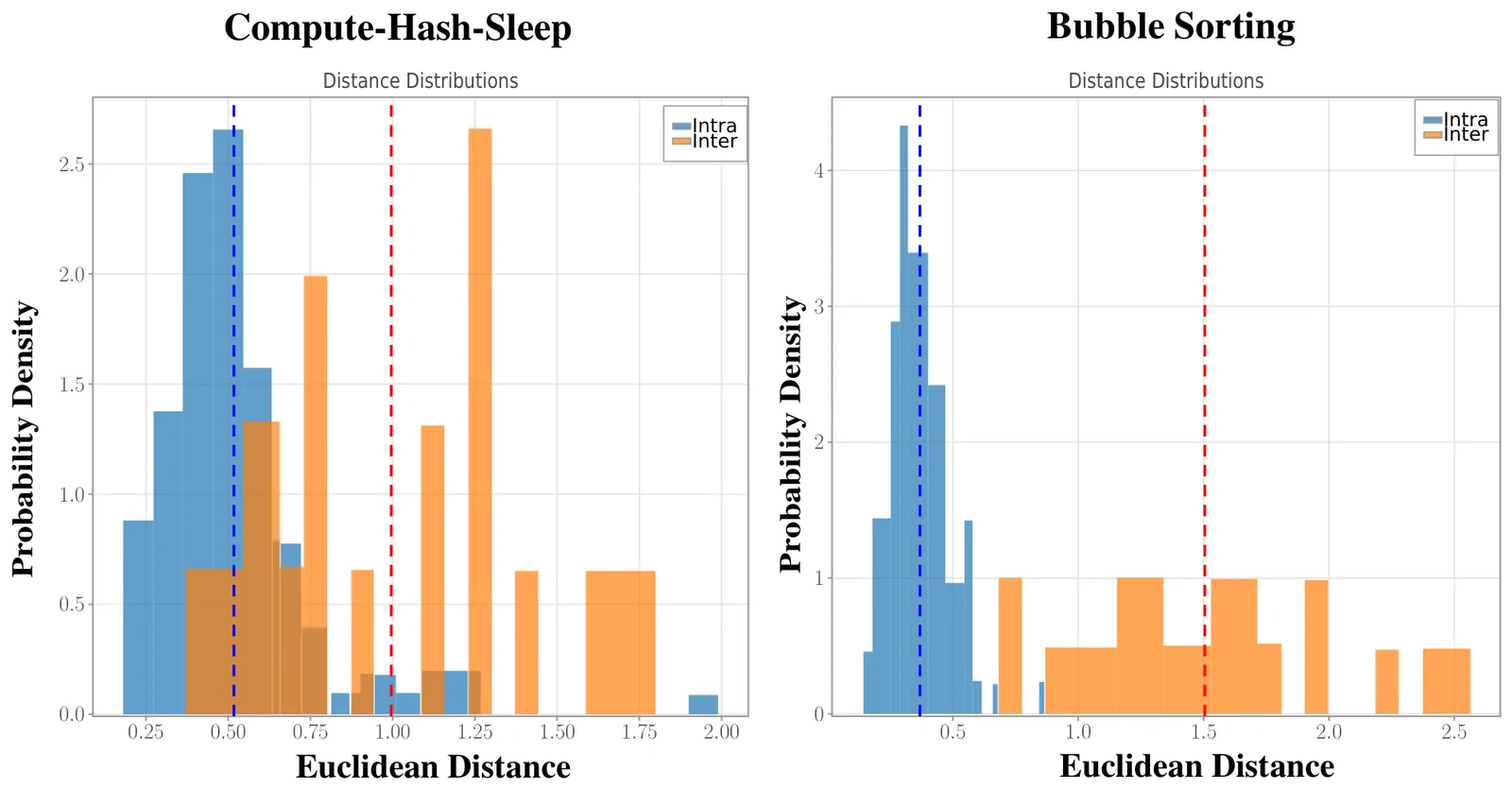
Workload Impact on Arduino Device Distinguishability.

To investigate how different workload characteristics affect device distinguishability, Arduino devices performed two tasks: Compute–Hash–Sleep, which emphasizes arithmetic operations, and Bubble Sorting, which introduces frequent comparisons, branching, and memory accesses. Table 4 demonstrates that Bubble Sorting improved the separation ratio 2.14× (4.10 vs. 1.92) and d-prime ($d^{\prime }$) 2.01× (3.18 vs. 1.58) compared to Compute–Hash–Sleep. Figure 8 illustrates these differences through distance distributions, showing that Bubble Sorting produces distinctly separated intra- and inter-device clusters. These results suggest that on this platform, the richer control-flow and memory-access patterns of Bubble Sorting lead to more distinctive power signatures than the primarily computational nature of hash-based workloads.

Intra-device clustering tightened from 0.517 to 0.367 (29% reduction), while inter-device distance expanded from 0.995 to 1.505 (51% increase). Classification performance improved across all six algorithms, with Bubble Sorting achieving 99.0–100.0% F1-scores (SVM and k-NN reached 1.00) compared to 62.0–68.0% for Compute–Hash–Sleep.

#### 5.1.3. Workload Impact on ESP32 Platform

ESP32 devices were tested with Compute–Hash–Sleep and Networked Sensor Monitoring workloads. Table 5 shows that the separation ratio remains high for both tasks (2.99 vs. 2.72, a 9.0% decrease), indicating that ESP32 devices remain well distinguishable even when the executed workload changes.

While intra-device distance tightened slightly for Sensor Monitoring (0.321 vs. 0.346) and inter-device distance compressed (0.876 vs. 1.036), the separation ratio decreased by 9.0%, from 2.99 to 2.72.

This high level of separability is reflected in the classification performance across six machine learning models, as detailed in Table 6. Random Forest achieved high performance for both workloads, with F1-scores of 95.5% for the hash loop and 96.2% for the sensor task—a negligible difference of only 0.7 percentage points.

While Logistic Regression and the RBF-kernel SVM demonstrated reduced performance on the more complex sensor workload, all classifiers maintained F1-scores above 80%, confirming that fingerprinting effectiveness on the ESP32 architecture remains robust across different computational tasks.

**Figure 9:**
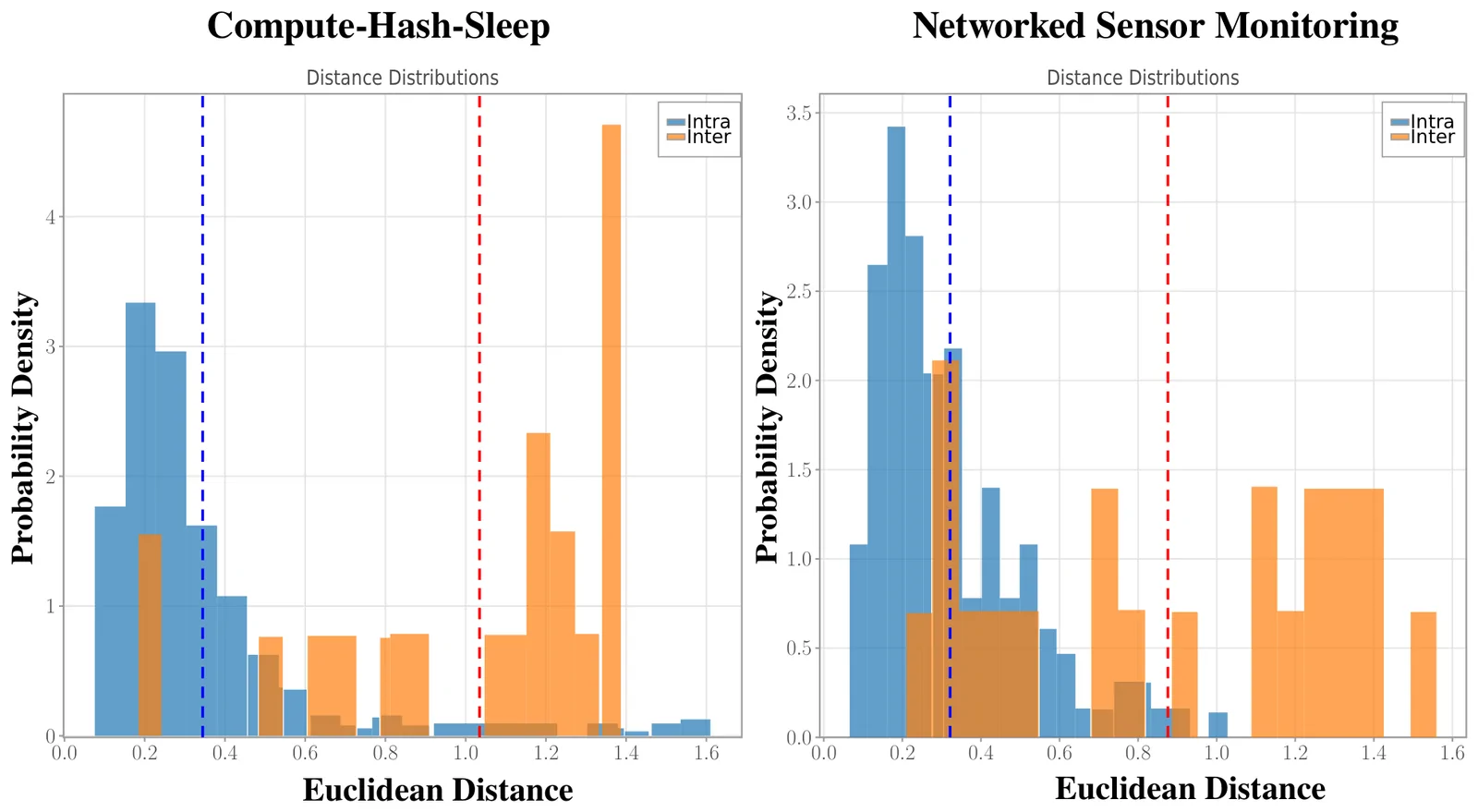
ESP32 Distance Distributions Across Workloads.

Figure 9 visualizes the distance distributions for both ESP32 workloads, showing how intra-device distances (blue) and inter-device distances (orange) shift between the Compute–Hash–Sleep and Networked Sensor Monitoring tasks.

### 5.2. Multi-Session Temporal Analysis (ESP32 Only)

The Networked Sensor Monitoring workload on the ESP32 platform was employed to assess power side-channel fingerprint temporal stability over the 45-day multi-session evaluation. ESP32 was selected for its superior device discriminability ($d^{\prime }$ = 2.54 vs. Arduino 1.25), which provides stronger baseline separability for reliably detecting temporal degradation in power features over time. The initial single-session analysis (each measurement session evaluated independently) demonstrated strong fingerprinting performance, with separation ratios ranging from 2.31 to 3.58 and F1-scores between 87.3% and 98.5% across six machine learning models. Strong within-session performance, however, does not carry across sessions.

When computing separation across all four sessions combined, the separation ratio collapsed to 0.988 (indicating near-zero between-device discrimination), and $d^{\prime }$ degraded to 0.0425. Statistical power analysis confirmed that temporal degradation exhibits large effect sizes (d=1.792 for d-prime, d=3.236 for separation ratio), demonstrating severe fingerprint instability. However, the moderate achieved power of 60% suggests that larger sample sizes would strengthen conclusions about long-term viability. These degradation patterns motivated the neural embedding approach evaluated next.

### 5.3. Neural Embeddings Performance

Evaluated under centroid-based cosine similarity scoring with percentile-based thresholding, the learned embeddings showed more consistent performance across the 45-day evaluation, unlike statistical features, which degraded notably. Our embedding approach retained 93.1% of initial performance (stability score 0.931) compared to 84.0% for raw features (stability score 0.840), providing a 9.1 percentage point advantage. Average embedding degradation across sessions was limited to 6.1%, while raw features degraded 14.5%—indicating embeddings maintained approximately 2.4× greater temporal stability in their feature representations (calculated as the ratio: 14.5%/6.1%). This improved stability is consistent with neural network learning to emphasize manufacturing-induced variations while suppressing temporal noise, though the precise mechanism was not isolated in this study.

To directly compare feature representations, we evaluated raw statistical features and neural embeddings using identical centroid-based cosine similarity scoring under three splitting protocols with percentile-based thresholds that balance false acceptance and false rejection rates. Table 7 presents the comparative results for genuine devices (TPR) and the three impostor scenarios defined in Section 3.4.4: unmodified non-enrolled devices (Real FAR), the quantization-and-noise perturbation (Hardware FAR), and the adversary scenario in which an impostor embedding is shifted toward the target with α=0.85 (Adversary FAR).

TPR represents the correctly identified enrolled devices; FAR represents unknown devices that are incorrectly accepted. Centroid-based scoring on embeddings achieved 93.9–95.0% TPR with 4.7–9.9% FAR across protocols, substantially outperforming the identical scoring rule applied to raw features, with TPR improvements of 22.3 percentage points (fixed split), 41.1 points (cross-validation) and 27.5 points (LORO).

Across all three protocols, embeddings consistently outperformed raw features. Most critically, in the LORO cross-session protocol—where test samples originate from sessions unseen during training—embeddings maintained 93.9% TPR with 9.9% FAR versus 66.4% TPR and 35.8% FAR for raw features (27.5 percentage point TPR advantage). Per-protocol breakdowns are detailed in Table 7. Confidence intervals in Table 8 confirm these results, with embeddings exhibiting consistently tighter intervals across all protocols. Because the operating threshold is calibrated independently for each held-out session, we further examined its cross-session stability. Expressed in per-fold impostor-normalized score units, the EER operating threshold varied across the four sessions with a within-device standard deviation between 0.18 and 1.02 across the seven devices (mean 0.56), while the FAR-optimized threshold was considerably more stable (mean 0.15). This residual variation is largely absorbed by the per-session impostor normalization, which is why verification performance is maintained despite session-to-session shifts in the underlying similarity scores.

The three impostor scenarios in Table 7 behave differently, and the contrast is informative. The quantization-and-noise scenario yields a FAR of 9.9% under the cross-session protocol, essentially identical to the realistic same-batch baseline (9.9%): because the perturbation in Equation (5) is small, these embeddings remain close to those of genuine non-enrolled devices and are rejected at the same rate. The adversary scenario is more demanding, raising the FAR to 26.8%. This elevation does not reflect a strong or physically realized attack—the perturbation in Equation (6) injects only a 15% component of the target—but rather indicates that the acceptance boundary, calibrated solely on genuine and unmodified impostor scores and held fixed, is looser against impostor embeddings shifted toward the target than against unmodified impostors. Even so, learned embeddings remain substantially more robust than handcrafted features under the same perturbation (26.8% versus 43.7% for raw features, Table 7). Tightening or loosening the operating threshold does not resolve this. At the FRR-optimized point, the adversary FAR falls to 20.9%, but the TPR drops to 82.9%, while the real and hardware FARs rise to 14.8% and 14.6%, respectively. At the FAR-optimized point, the adversary FAR remains 25.3%, and the TPR is reduced to 81.1%, even though this threshold is explicitly chosen to reject impostors. Neither operating point removes the vulnerability; both merely move along the same trade-off curve. The principled mitigation is therefore not threshold adjustment but representation-level hardening—adversarial training in which synthesized adversary embeddings are introduced as hard negatives so that the network itself learns to separate them from genuine centroids—which we identify as future work (Section 6.4, Adversarial Robustness) and which our metric-learning objective naturally accommodates. Because this scenario additionally presumes knowledge of the target representation and the ability to operate directly in embedding space, protecting the measurement and representation pipeline (Section 6.3) further raises the barrier to mounting it.

The protocol also admits a variant in which the impostor identity itself is withheld. Each of the seven devices is removed from the pool in turn: the encoder is trained on the remaining six, those six are enrolled and supply genuine trials, and every window of the withheld device is presented as an impostor drawn from an identity absent from both the training data and the classification head. The LORO structure is retained within each fold, so identity and session are withheld together, and both representations are evaluated on the same folds under the same scoring rule. Across all seven positions and four sessions (168 device-folds), embeddings sustain a 91.1% true positive rate at a 26.0% false acceptance rate against unseen devices (95% CI [20.7, 31.7]), while statistical features under the identical protocol reach 66.3% and 39.0% (95% CI [32.3, 45.9])—an advantage of 24.8 percentage points in true positive rate and 13.0 points in false acceptance. As a control, the enrolled-impostor rate measured in these same runs is 10.4%, consistent with the 9.9% of Table 7; the change therefore reflects the withheld identity rather than the smaller enrolled pool, which differs by six devices rather than seven. The corresponding true positive rate, 91.1% against 93.9%, likewise tracks the smaller pool.

The size of that difference follows from where the operating threshold is placed. Calibration draws on genuine scores together with impostor scores from devices the angular margin has explicitly separated, so an identity never subject to that margin is not displaced from the target centroid, and a fixed boundary admits it at a rate comparable to the adversary scenario above, where the perturbation likewise places the impostor outside the region the margin acts on. The remedy identified there applies equally: introducing withheld or synthesized identities as hard negatives during training acts on the representation rather than on the threshold, and the metric-learning objective accommodates this directly.

**Figure 10:**
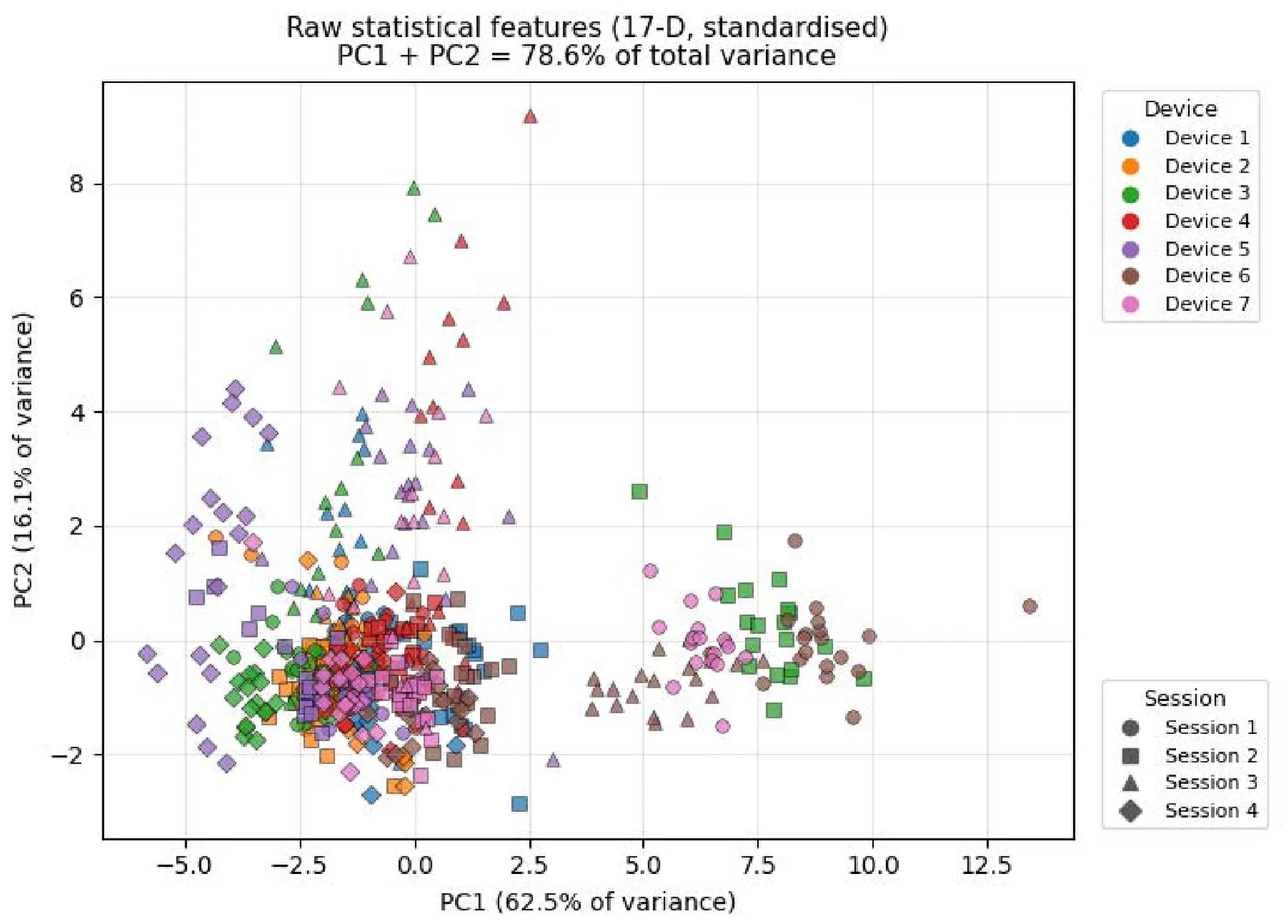
Principal-component projection of the 17-dimensional statistical feature vectors for all 532 windows (7 devices × 4 sessions × 19 windows), standardized to unit variance before projection. Color denotes device and marker shape denotes measurement session. PC1 accounts for 62.5% and PC2 for 16.1% of the total variance (78.6% combined). The dominant structure is session-aligned: windows group by acquisition session, with devices intermixed within each group.

**Figure 11:**
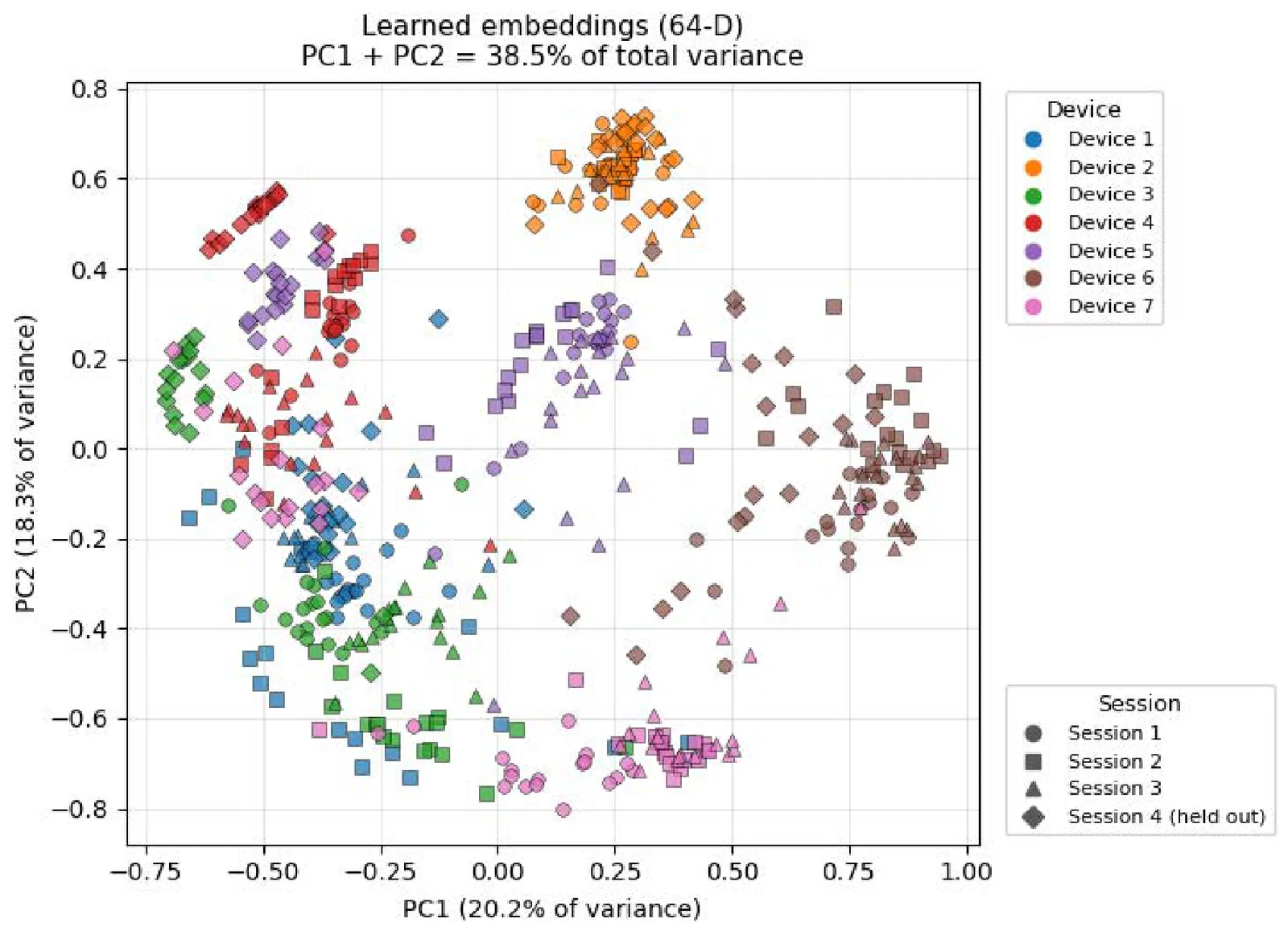
Principal-component projection of the 64-dimensional learned embeddings for the same 532 windows, using the same color and marker encoding as Figure 10. PC1 accounts for 20.2% and PC2 for 18.3% of the total variance (38.5% combined). Here the dominant structure is device-aligned: each device forms a compact cluster containing windows from all four sessions.

Figure 10 and Figure 11 visualize the difference between the two representations using Principal Component Analysis (PCA) [49] for dimensionality reduction, with device encoded by color and measurement session by marker shape so that both sources of structure are visible simultaneously.

The two projections are organized along different axes. In the statistical feature space, the leading components separate the acquisition sessions: windows recorded in the same session group together while devices remain intermixed within each group, so the strongest signal in the representation is when a measurement was taken. In the embedding space, the ordering reverses. Each device occupies a compact region containing windows from all four sessions together, and session membership is no longer the organizing variable. The network has learned to map traces into a space in which same-device windows converge and different-device windows separate, and the session-to-session variation that dominates the statistical features is substantially suppressed. These are two-dimensional summaries of a 64-dimensional space, and the quantitative counterpart is the LORO result of Table 7; read together, they show the same effect from two directions.

### 5.4. Statistical Power Analysis

Aggregated results from all test samples (35 embeddings versus 35 raw features) show that neural embeddings deliver consistently higher performance when distinguishing batch-identical devices in intrusion detection scenarios. The observed effect size (Cohen’s d=0.774) and 89.1% statistical power comfortably exceed the conventional 80% reliability threshold, validating that the dataset from seven devices provides adequate sample size for detecting meaningful differences.

Further analysis at the individual device level revealed that embeddings perform well for most devices, though some variability exists. Six devices (0, 1, 3, 4, 5, 6) reached excellent statistical power (≥96%, Cohen’s *d* ranging from 3.2 to 9.1), while Device 2 showed substantially lower statistical power (30.6%, Cohen’s d=1.357). We emphasize that this is a measure of statistical confidence rather than recognition performance: Device 2’s low power reflects a single anomalous fold in which impostor rejection failed, while its remaining three folds rejected all or nearly all impostors and genuine samples were accepted in every fold.

**Figure 12:**
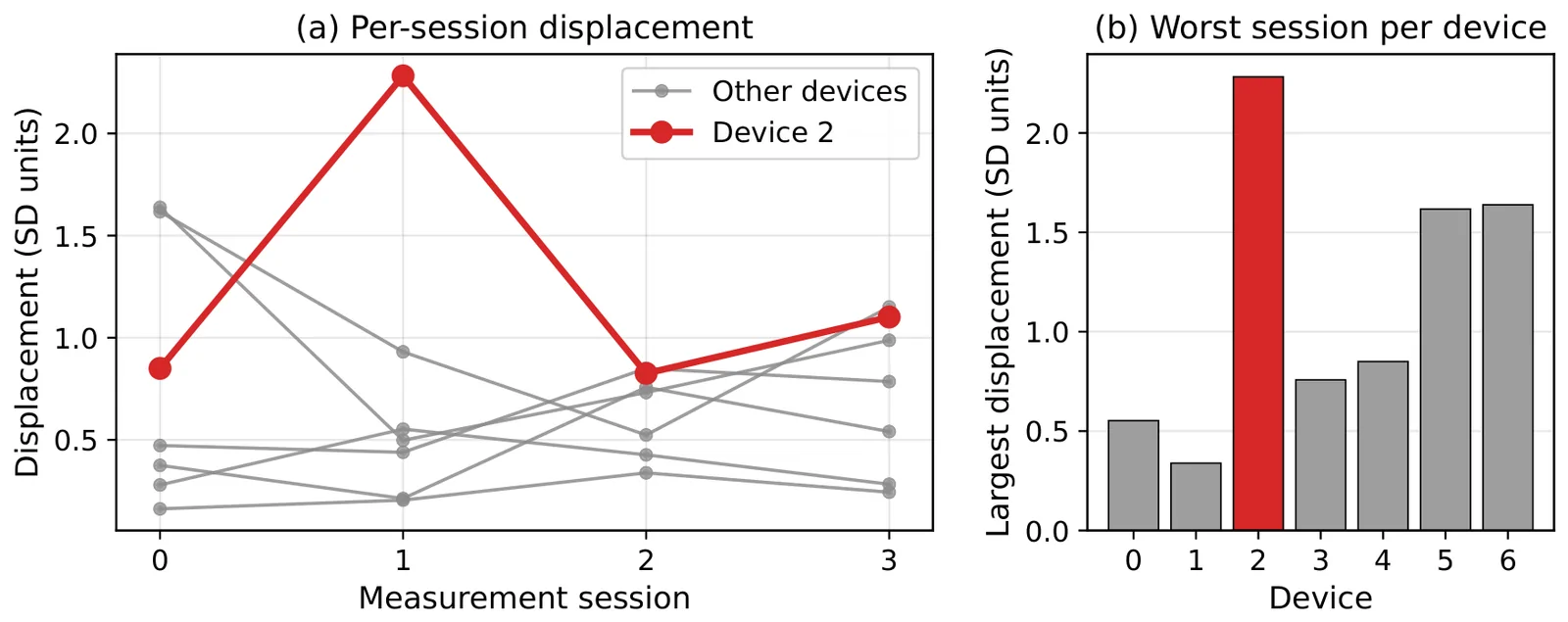
Session-to-session behavior of all seven ESP32 devices over the 45-day evaluation. (**a**) For each device and session, the displacement of that session’s mean feature vector from the same device’s other three sessions, expressed in standard deviations of the pooled cross-device feature distribution. (**b**) The largest such displacement for each device. Isolated session-level excursions occur throughout the pool—worst-session values range from 0.34 to 1.64 standard deviations across the other six devices—indicating that session-to-session variability is a property of the acquisition rather than of any individual unit. Device 2’s session 1 is the largest excursion in the study at 2.28 standard deviations, twice that device’s own next-largest session.

We traced this to a single measurement session for that device. That session’s mean feature vector is displaced from Device 2’s own three remaining sessions by 2.28 standard deviations of the pooled cross-device feature distribution, against 0.83–1.10 for its other sessions, and the shift is distributed across the feature set rather than confined to one descriptor: averaged over all seventeen features, the session’s windows lie 1.91 standard deviations from the distribution of the other six devices, compared with 0.70–0.91 for the same device’s remaining sessions. This is the signature of an acquisition-level effect in that recording rather than of an intrinsic device property, and a controlled retraining test confirms the attribution. The affected session is not the one held out when verification failed; it forms part of the training set for that fold. Withholding it from training reduces Device 2’s false acceptance in the failed fold from 0.82 to 0.40 while raising its genuine acceptance from 0.70 to 0.90, whereas withholding a different session as a control leaves false acceptance at 0.95. Figure 12 places this session against the session-to-session behavior of all seven devices. Two other devices show comparable single-session excursions, of 1.62 and 1.64 standard deviations, yet reach 100% and 96.2% statistical power; displacement alone therefore does not determine per-device confidence, and it is the retraining test that isolates the effect for Device 2. With only four sessions per device, such a single affected session exerts an outsized influence on per-device statistics.

Across all devices, the mean power was 89.5%, with 85.7% meeting or exceeding the reliability standard of 80%. These results indicate that, while per-device statistical confidence is occasionally limited by session-level measurement variability, neural embeddings generally outperform raw features for hardware fingerprinting.

Statistical power for the temporal comparisons is more limited: the achieved power of 60% reported in Section 5.2 indicates that, while the temporal degradation itself is reliably detectable given its large effect sizes, the multi-session design does not support equally confident conclusions about the magnitude of the embedding advantage over time. While trends do favor embeddings in temporal stability, future studies need finer isolation of environmental variables and larger session counts for more definitive conclusions (Section 6).

### 5.5. Ablation Analysis

To identify which components of the embedding pipeline drive the observed temporal robustness advantage, we conducted a systematic ablation study. Each experiment removed or modified a single component while keeping all others at their default configuration, using the LORO cross-session protocol with EER-based thresholds. Table 9 presents the results.

The ablation reveals a clear hierarchy of component importance. AM-Softmax angular margin enforcement is the critical loss component: removing it (triplet-only configuration) causes a 12.5 percentage point TPR reduction—the largest single-component effect. In contrast, removing triplet loss while maintaining AM-Softmax leaves the true positive rate unchanged and shifts false acceptance only slightly, from 11.7% to 11.3%. The identical true positive rate is a resolution effect as well as a substantive one: the Leave-One-Run-Out protocol provides 28 device-folds of ten test windows each, so this rate is quantized to multiples of 1/280 and both configurations accept the same 264 of 280 genuine windows. False acceptance, which is measured over six times as many trials per fold, resolves the small residual difference between them. Taken with the 12.5-point loss when AM-Softmax is removed instead, this indicates that the angular-margin term supplies the separation on which open-set performance depends, while the triplet term is not contributing separably once that margin is present—consistent with both acting on the same geometry, one through an explicit angular margin and the other through relative distances. Data augmentation and the choice of cosine versus Euclidean scoring similarly have negligible impact.

The residual connection and batch normalization each contribute moderately, with 5.0- and 4.6-percentage-point TPR reductions, respectively, when removed. Embedding dimensionality shows diminishing returns: reducing from 64D to 32D has a minimal effect (−0.7%), while increasing to 128D slightly degrades performance (−2.5%), suggesting potential overfitting with higher dimensionality.

These results identify angular margin enforcement as the primary mechanism enabling temporal robustness. Residual connections and batch normalization provide secondary but meaningful contributions, while triplet loss, data augmentation, and scoring metrics do not measurably affect open-set detection performance.

Having established which components drive temporal robustness, we now assess deployment feasibility.

### 5.6. Deployment Feasibility and Scalability Analysis

#### 5.6.1. Model Architecture and Parameter Budget

The inference-only component of the embedding network—the residual encoder excluding the AM-Softmax classification head—comprises 78,912 parameters organized as a 17→256→128→64 fully connected stack with batch normalization and one residual shortcut. Parameter storage in IEEE 754 [50] single precision (float32) is 308.2 KB; quantizing weights to eight-bit integers (int8) reduces this to 77.1 KB. These two figures are the theoretical quantized and unquantized parameter payloads. When the network is compiled to a TensorFlow Lite Micro flatbuffer and deployed on the ESP32-WROOM-32 used throughout this study, the resulting on-Flash artifacts are larger, since they additionally embed the model container, operator table, and quantization metadata: the deployed int8 artifact occupies 95 KB (97,520 bytes) and the float32 artifact 310 KB (318,160 bytes). At run time, inference executes within a fixed 64 KB tensor arena allocated in SRAM—a working-memory requirement separate from, and not additive to, the Flash storage figures above. Each enrolled device contributes one 64-dimensional float32 centroid vector (256 bytes). Total memory at deployment scales as

(11) $$M\left(n\right)=M_{\mathrm{model}}+n\cdot m_{c}$$

where $M_{\mathrm{model}}=308.2$ KB (float32) or 77.1 KB (int8), $m_{c}=256$ bytes per centroid, and *n* is the enrolled device count. As shown in Table 10, the stored system fits within the ESP32’s 4 MB Flash at every scale evaluated. Against the tighter 520 KB SRAM budget, the float32 configuration remains resident up to 500 devices (433.2 KB) but exceeds it from 1000 devices onward (558.2 KB), whereas int8 quantization extends residency to approximately 1000 devices (327.1 KB). Beyond that scale, neither precision holds the full model-plus-gallery footprint in SRAM, and the centroid table would be retained in Flash and streamed during scoring.

#### 5.6.2. Inference Latency

Embedding inference was benchmarked on CPU (x86_64, PyTorch 2.3.1) over 500 independent trials, with the model pinned to the same device as the input tensor and 10 warm-up forward passes discarded before timing. The mean embedding time is 0.169±0.020 ms. The total per-query computational cost decomposes as

(12) $$T_{\mathrm{query}}\left(n\right)=T_{\mathrm{embed}}+n\cdot t_{c}$$

where $T_{\mathrm{embed}}=0.169$ ms is the fixed embedding cost and $t_{c}$ is the per-centroid cosine scoring time. Gallery scoring is a pure NumPy operation on CPU. Table 11 reports measured values across gallery sizes. Scoring 500 centroids adds only 0.104 ms, keeping total query latency below 0.28 ms—well within the requirements of online monitoring applications. The linear growth in Equation (12) is confirmed empirically across the tested range (7–5000 centroids), validating the computational model. These figures are reference benchmarks on commodity hardware rather than on-device measurements: repeating the same benchmark on a Tesla T4 GPU yields 0.367±0.051 ms embedding time, slightly slower than the CPU because kernel-launch overhead dominates for a network this small. Because the intended deployment target is a microcontroller whose architecture differs substantially from an x86_64 CPU, we measured its on-device latency directly rather than inferring it from these commodity figures, as reported next.

We deployed the trained encoder to the ESP32-WROOM-32 (Xtensa LX6 dual-core, 240 MHz) used throughout this study via TensorFlow Lite Micro, with the final L2-normalization computed in C++ outside the TensorFlow Lite graph. On this target, int8-quantized embedding inference runs in 8.88 ms (8880 µs mean over 4160 samples; standard deviation 0.84 µs; min/max 8878/8904 µs), approximately 112 inferences per second. The float32 reference model runs in 15.44 ms (mean over 509 samples; standard deviation 1.90 µs), approximately 64 inferences per second. Quantization therefore yields a 1.74× speedup and a 3.26× reduction in on-Flash artifact size (310 KB →95 KB), while both precisions execute within the same 64 KB SRAM tensor arena. This 8.88 ms latency is the encoder forward pass, from a prepared feature vector to an L2-normalized embedding. Placed in the context of a complete verification, it is the smallest of the three costs involved: gallery scoring adds 128 floating-point operations per enrolled device, feature extraction is a single pass of order statistics and one real FFT over a window, and trace acquisition itself takes on the order of seconds for the window lengths used here. Verification latency in deployment is therefore set by acquisition rather than by inference, which places on-device embedding comfortably within the periodic-verification model assumed throughout this work.

We additionally re-validated detection accuracy at int8 precision. Quantizing the encoder’s weights and activations to 8-bit integers, with the representative set drawn from the training and validation partitions of each fold, and re-scoring the Leave-One-Run-Out test partitions under the same balanced-threshold rule gives 92.5% TPR at 10.1% FAR, against 93.6% TPR at 9.6% FAR for the identical float32 weights on the same partitions. The two precisions differ by 1.1 and 0.5 percentage points, respectively—three of the 280 genuine windows and eight of the 1680 impostor comparisons—so quantization preserves detection performance to within approximately one percentage point on this platform.

As an independent cross-check, this latency is consistent with the order-of-magnitude expectation set by the model’s 157,376-FLOP profile and by published TensorFlow Lite Micro benchmarks on comparable embedded targets [51,52], which place inference for networks of this scale in the low-milliseconds to tens-of-milliseconds range. This serves only as a sanity check; the on-device measurement is the figure we report for our platform. Latency and memory are now measured directly; on-device energy profiling, together with the resulting trade-off between verification frequency and power consumption on low-power sensor nodes, remains for future work (Section 6.4).

#### 5.6.3. FLOPs and Computational Complexity

The embedding forward pass requires 157,376 floating-point operations; each cosine similarity comparison against one centroid adds 128 FLOPs. Total per-query cost therefore scales as 157,376+128n, growing from 158,272 FLOPs at seven enrolled devices to 797,376 at 5000—roughly a five-fold increase. The fixed embedding term dominates for galleries up to approximately 1200 devices; beyond that point, linear gallery scoring becomes the larger contributor. In absolute terms, however, even the 5000-device query remains below 0.8 MFLOP, so verification stays lightweight across the entire evaluated range.

#### 5.6.4. Embedding Space Geometry and Scalability Projection

Beyond computational cost, practical scalability requires that device centroids remain geometrically distinguishable as the enrolled population grows. We characterize the 64-dimensional embedding space using two complementary analyses.

##### Observed Geometry (7 Enrolled Devices)

The seven trained centroids lie on the unit $L_{2}$-norm hypersphere in $R^{64}$. Pairwise cosine similarities range from −0.651 (most separated pair) to +0.208 (closest pair), corresponding to angular separations of $130.6^{\circ }$ and $77.9^{\circ }$ respectively, with a mean of $97.0^{\circ }$. The random-vector baseline in $R^{64}$ predicts E[cosθ]=0 with σ≈0.125 (i.e., random vectors are expected near-orthogonal), so the observed mean angle of $97.0^{\circ }$ confirms that trained centroids are well-distributed and not collapsing toward any common direction.

##### Scalability Simulation

To project geometric behavior beyond seven devices, we employ a simulation seeded by the measured centroids. Let ${\left\{\mu_{i}\right\}}_{i=1}^{7}$ denote the real centroids. The perturbation spread is estimated from the data as

(13) $$\sigma_{\mathrm{perturb}}=\mathrm{std}\;\left(\mu_{i}-\bar{\mu }\right),\;\bar{\mu }=\frac{1}{7}\sum_{i=1}^{7}\mu_{i}$$

yielding $\sigma_{\mathrm{perturb}}=0.1228$, consistent with the theoretical expectation of $1/\sqrt{64}=0.125$ for well-spread embeddings. Synthetic centroids for additional devices are generated as

(14) $${\tilde{\mu }}_{j}=\frac{\mu_{b(j)}+\varepsilon_{j}}{∥\mu_{b(j)}+\varepsilon_{j}{∥}_{2}},\;\varepsilon_{j}∼N\left(0,\;\sigma_{\mathrm{perturb}}^{2}I\right)$$

where b(j) is a real centroid drawn uniformly at random. Re-normalization in Equation (14) projects the perturbed vector back onto the unit hypersphere, preserving the cosine-similarity geometry used at inference time. The value $\sigma_{\mathrm{perturb}}$ is computed directly from the experimental centroids rather than assumed a priori, making the simulation data-informed. We assume that unseen device centroids follow the same distributional spread as the enrolled population; this is conservative in the sense that greater diversity in a larger pool would compress separations faster than simulated, placing the simulation on the optimistic side for larger *n*. In particular, additional devices drawn from the same manufacturing process may populate the embedding space more densely than independent perturbations of seven centroids suggest, so the projected minimum separations should be read as optimistic estimates pending empirical validation at scale.

**Figure 13:**
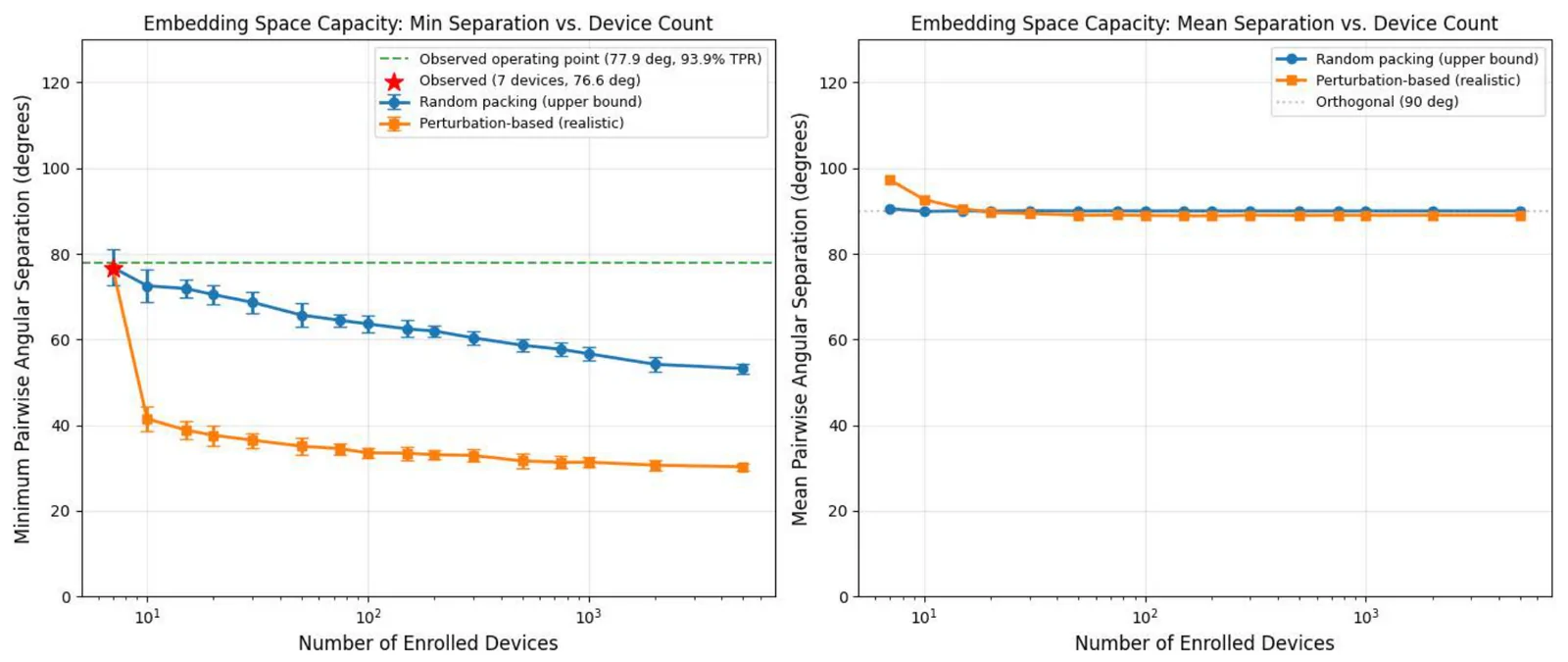
Embedding space capacity simulation. (**Left**): minimum pairwise angular separation versus enrolled device count for random-packing (upper bound, blue) and perturbation-based (data-conditioned estimate, orange) modes. The green dashed line marks the observed 7-device operating point (77.9°, corresponding to 93.9% TPR at 9.9% FAR). (**Right**): mean pairwise separation remains near $90^{\circ }$ across the full range, confirming that global hypersphere geometry is preserved even as the minimum compresses. The n=7 point is measured; larger populations are generated under the perturbation model.

To bound the result from above, we additionally run a random-packing simulation in which synthetic centroids are drawn as independent uniform unit vectors, representing the best achievable separation for any learned embedding. The two curves together bracket the expected minimum pairwise angular separation as a function of enrolled device count (Figure 13).

**Figure 14:**
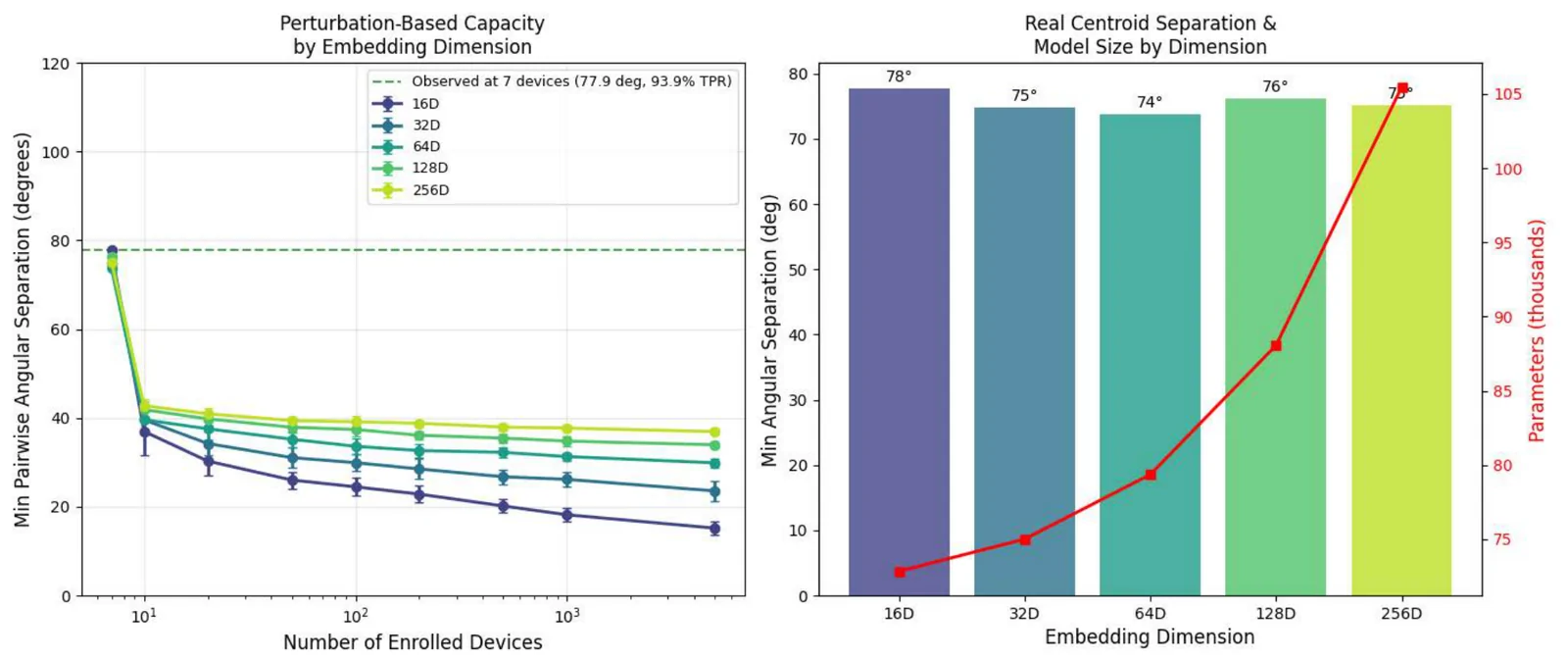
Effect of embedding dimension on geometric capacity and model size. (**Left**): perturbation-based minimum angular separation versus device count for d∈{16,32,64,128,256}. Higher dimensions preserve separation at larger device counts at the cost of increased parameter count. (**Right**): observed 7-device minimum separation (bars) and parameter count (red line) across dimensions; all dimensions achieve >73° at 7 devices with marginal separability benefit above 64D. Values at 7 devices are measured; those at larger counts are generated under the perturbation model.

Figure 14 and Table 12 summarize minimum angular separation at seven and 5000 devices for each embedding dimension, each measured on a network trained at that dimension. At the observed seven-device operating point (77.9°), the system achieves 93.9% TPR at 9.9% FAR. As the simulated population grows, minimum separation decreases and stabilizes at a dimension-dependent floor: approximately 15° for 16D, 24° for 32D, 30° for 64D, 34° for 128D, and 37° for 256D at 5000 devices. The relationship between angular separation and recognition accuracy at these reduced margins cannot be determined from the current seven-device dataset; establishing this mapping requires empirical evaluation with larger device pools. The 64-dimensional embedding used in this work requires only 79,360 parameters (310 KB), while 128D and 256D offer higher geometric floors at modest cost (88K and 105K parameters, respectively), providing a design lever for deployments requiring larger device populations.

This analysis characterizes how the geometry of the embedding space evolves as the enrolled population grows. The seven measured centroids anchor it at n=7; larger populations are generated from them under the perturbation model of Equation (14), and the two simulation modes bracket the range of packings a learned embedding can be expected to produce. What the curves establish is that the space retains usable angular margin as the population scales, and that embedding dimension is the design lever that governs the floor. Relating a given angular margin to a detection rate at those larger populations is a measurement question, and extending the device pool to answer it is a natural next step for this line of work.

## 6. Discussion

### 6.1. Interpretation of Key Findings

The results demonstrate that temporal degradation is not a secondary effect but a defining factor for deployment viability. The two representations compared here are derived from the same recorded traces and evaluated on the same folds under the same scoring rule, so whatever conditions prevailed during each session applied identically to both. Any difference in their temporal behavior is therefore attributable to the representation itself rather than to the circumstances of measurement. The size of that difference is reported in Section 5.2 and Table 7: the separation afforded by the statistical features falls away once sessions are pooled, while the embeddings sustain verification on sessions withheld from training. Representation choice is on this evidence a first-order design factor for temporal robustness, to be settled when a system is designed rather than compensated for after it is deployed.

ESP32 achieves a higher $d^{\prime }$ (2.54 vs. 1.25) and stronger classification performance than Arduino under identical conditions, as detailed in Section 5.1.1. Architectural complexity is one plausible explanation: additional subsystems provide more circuit blocks in which manufacturing variations can manifest.

The temporal robustness results (Section 5.2) confirm that learned embeddings provide more stable device-identity information than statistical features across sessions. Statistical features capture only global distribution properties, making them vulnerable to environmental drift and measurement noise. Ablation analysis (Table 9) confirms that AM-Softmax angular margin enforcement is the critical factor, as detailed in Section 5.5. This suggests that enforcing angular separation between device classes contributes more to temporal stability than minimizing intra-device variation, as commonly emphasized in metric learning.

While the 45-day evaluation demonstrates that neural embeddings sustain device discrimination under controlled laboratory conditions, the stability of power-based fingerprints across realistic environmental variations remains unexplored. Our results provide proof-of-concept evidence but motivate systematic investigation of environmental factors—temperature, humidity, supply voltage, and aging—that may degrade fingerprint robustness. Isolated testing of each factor will clarify which primarily drive fingerprint drift. Combined with longer operational periods and field deployment, such controlled experiments are essential to translate feasibility into deployment-ready mechanisms for practical IoT security.

### 6.2. Metric Learning for Power Side-Channels

While metric learning objectives such as triplet loss and angular-margin Softmax are well established in person re-identification [40] and face verification [41], their relative contributions have not been evaluated in the power side-channel domain. Most prior power side-channel fingerprinting studies relied on standard classification losses (e.g., Cross-Entropy), which optimize for separability only within a closed training set. Our ablation analysis (Section 5.5) shows that AM-Softmax angular margin enforcement alone is sufficient for robust open-set recognition. This has practical implications: deployments can omit triplet mining without sacrificing temporal robustness.

### 6.3. Practical Deployment Considerations

The LORO cross-session result (93.9% TPR, 9.9% FAR, Table 7) provides proof-of-concept evidence for power side-channel fingerprinting for device discrimination under controlled laboratory conditions. Assuming power measurement infrastructure can be adequately protected, power side-channel fingerprinting could complement multi-factor authentication systems in scenarios where devices are enrolled under organizational control. A two-tier arrangement suits this profile, with power side-channel verification acting as a fast first stage and secondary authentication handling the cases it does not clear. The achievable composite error rate then depends on the second factor chosen and on its independence from the power measurement chain, which is a question for a dedicated deployment study.

Moving from these laboratory results toward deployment raises the question of how the assumption of trusted measurement infrastructure in Section 3.1 could be enforced in practice. What must be trusted lies entirely on one side of the measurement. Acquisition, scoring and the stored reference all sit on the monitoring unit, while the device under test contributes only its own supply current as it re-executes the enrollment workload, running no verification code and holding no reference—so a fingerprint reported by a device about itself is bounded by the integrity of that device’s firmware, whereas an externally measured supply current is not. Securing the monitoring unit is therefore the whole of the problem, and one approach is to run the measurement chain—trace acquisition, storage of the device references, and similarity scoring—inside a Trusted Execution Environment (TEE) on the monitoring unit [53]. A TEE isolates this code from the rest of the host so that a compromised host cannot alter the captured traces, the stored references, or the verification result, and can also produce signed evidence that the result came from the unmodified verification code. A second concern is the reuse of an old, recorded measurement of a legitimate device in place of a fresh one. This can be prevented by having the verifier issue a fresh random value for each verification and requiring the measurement unit to return its reading together with that value and a timestamp, signed, so that readings captured in an earlier session no longer pass. This safeguard changes only how a measurement is requested and reported, leaving the workload itself unchanged and preserving the deterministic execution conditions under which our results were obtained. Acquisition would likewise remain controlled in the field, since verification is performed in dedicated measurement windows in which the device re-executes the enrollment workload on the in-line monitor, consistent with the periodic-verification model assumed throughout this work. Environmental stability is the one assumption that such mechanisms cannot address and remains the principal open question for deployment, as discussed in Section 6.4. The measures described here are architectural proposals intended to guide future deployment work rather than mechanisms evaluated in this study.

Because verification re-executes the workload used at enrollment, the fingerprinting task is a deployment choice rather than a fixed requirement: in a heterogeneous network, a node’s own application task can serve as it. How much that choice matters differs between the two platforms. On ESP32, the two workloads tested lie at opposite ends of the device’s activity range—computation with deep-sleep cycles, and sensor acquisition with wireless transmission—yet the separation ratio differs by only 9% between them (Table 5), so a node can be fingerprinted on whichever of them it runs. On Arduino, the task has a larger effect, with memory-intensive Bubble Sorting amplifying discriminability well beyond arithmetic (Table 4, $d^{\prime }=3.18$ vs. 1.58), which makes the choice of verification task a design decision on that platform.

### 6.4. Limitations and Future Work

**Temporal Scope:** Our 45-day evaluation captures operational variability but represents only a fraction of typical IoT device lifetimes (3–5 years). Component aging—threshold voltage shifts from negative bias temperature instability, hot carrier injection, and electromigration—accumulates gradually over years and may alter fingerprints unpredictably. Over a multi-year span, such aging could in principle shift a device’s signature far enough from its enrolled reference to cause rejection of a legitimate device, or, less likely, to blur the separation between devices; neither effect is observable within our 45-day measurement period, so whether aging eventually degrades fingerprint validity remains an open question that periodic re-enrollment may need to address.

**Deployment Conditions:** Our experiments were conducted in a laboratory under nominally consistent ambient conditions, which do not reproduce the range encountered in field deployments. Ambient conditions were not logged, so the session-to-session component of any environmental variation cannot be separated from other time-variant effects; within a session, such variation acts on all devices alike and therefore does not bias them relative to one another. Isolating it would require controlled environmental logging alongside acquisition. Industrial IoT environments pose further challenges, including temperature extremes, electromagnetic interference from motors and RF equipment, and unstable power supplies. Future work should therefore evaluate robustness under controlled conditions that systematically vary these factors—including (1) temperature cycling (−20 °C to +60 °C), (2) electromagnetic interference per IEC 61000-4 standards [54], and (3) power supplies with voltage ripple and transients—over studies of at least 6–12 months, which would be long enough to separate environmental drift from the aging effects noted above. Adaptive thresholds that account for known environmental conditions could enhance reliability, and the 60% statistical power achieved for the temporal comparison underscores the need for these larger, controlled studies. Independently of whether the source of session-to-session drift is environmental, instrument-related, or due to aging, periodic re-enrollment—re-training each device’s reference embedding on freshly captured traces—re-anchors the stored fingerprint to current operating conditions and provides a practical mechanism for bounding such drift between enrollment intervals.

**Measurement Fidelity:** A single Arduino board, whose on-chip ADC provides 10-bit resolution, served as the measurement unit for all devices across all sessions. We adopted this shared acquisition path so that recorded differences would reflect the devices being tested rather than variation between instruments. The resolution and noise of this one measurement chain are therefore present in every trace, and their isolated effect on the learned representations was not separately quantified. Within a single session, this is not a source of error in device discrimination: because the same unit measures every device, its resolution limit and noise appear equally across the pool, so devices are distinguished because they genuinely differ and not because of the instrument. Across sessions, the situation differs: the recorded signatures are shifted from one session to the next by several time-dependent factors—ambient environmental conditions, gradual device aging, and drift in the measurement unit. This variation is what degrades the handcrafted statistical features over the 45-day period. The embedding network, however, is trained on data spanning all sessions, so it learns to factor out this shared session-to-session variation while preserving per-device identity. The temporal results in Section 5.2, where embeddings degrade far less than statistical features under the identical measurement chain, are consistent with this: whatever drift the single unit contributes over time is largely accommodated by the learned representation. One aspect this design cannot isolate is the effect of ADC resolution itself, which is fixed for a given unit and therefore identical across every trace; we state this as a bound on what the present single-instrument study can separate rather than as a measured quantity. The instrument’s own session-to-session drift is bounded in the same way: with no independent reference recorded alongside the devices, it remains grouped with the environmental and aging components identified above rather than resolved against them. A stable reference load acquired at the start of each session would supply that separation, since any displacement observed in a signal of fixed amplitude is attributable to the acquisition chain alone and can be removed from the device traces before analysis. Incorporating one is a direct extension of the present protocol and would convert the bound stated here into a measured quantity.

**Scalability and Generalizability:** Our seven-device dataset represents a proof-of-concept scale. Field trials extending 6–12 months across temperature ranges (−20 °C to +60 °C) and humidity variations (10–90%) are essential before production deployment.

Testing devices from multiple manufacturing batches, production sites, and vendors will validate whether our approach generalizes beyond this single-batch scenario. The variability we observed (Device 2: 30.6% power vs. others ≥96%) reflects session-level measurement variability rather than a manufacturing defect (Section 5.4), and characterizing how often such variation arises would benefit from larger multi-batch studies. Future work should determine optimal device pool sizes for different accuracy targets (95% vs. 99% TPR) through simulation-based power analysis. We do not claim that results generalize to all IoT microcontrollers. Platforms with different process nodes (e.g., 7 nm vs. 180 nm), power management architectures (e.g., ARM Cortex-M with Dynamic Voltage and Frequency Scaling (DVFS)), or analog front-ends may exhibit different fingerprint characteristics. Evaluating architectures between the two extremes tested here—such as 32-bit ARM Cortex-M devices with hardware cryptographic accelerators and dynamic voltage scaling—would clarify whether fingerprint quality scales with architectural complexity or plateaus beyond a certain design threshold.

**On-Device Deployment:** The embedding network has now been deployed to the ESP32-WROOM-32 via TensorFlow Lite Micro, and its inference latency, memory footprint, and int8 detection accuracy were measured directly (Section 5.6): 8.88 ms per int8 embedding within a 64 KB SRAM arena. This converts the earlier analytical estimate into measured values and establishes on-device feasibility. Energy-per-inference profiling and the associated trade-off remain future work: because continuous fingerprinting competes with a node’s primary workload for a limited power budget, how often and how thoroughly a device is re-verified must be balanced against power consumption on battery- or energy-harvesting nodes, which we regard as an important dimension of practical deployment.

**Adversarial Robustness:** Our threat model assumes the presence of passive attackers deploying cloned devices, not active adversaries crafting power profiles to mimic legitimate signatures. This gap remains unexplored in hardware fingerprinting. Sánchez Sánchez et al. [55] provide the closest evidence: a hardware-behavior identification model trained on 45 identical single-board devices proved unaffected by ambient temperature manipulation, yet was successfully bypassed by standard evasion attacks, with adversarial training combined with model distillation emerging as the most effective defense. Future research should evaluate (1) spoofing attacks using generative models trained on captured traces, (2) replay attacks reusing legitimate signatures, (3) adversarial training to improve robustness, and (4) measurements from deliberately manufactured near-clone devices, which would let the perturbation strength of Equation (6) be tied to an achievable cloning capability rather than set as a sensitivity parameter. Our metric learning framework naturally extends to adversarial triplet generation during training. Securing the measurement chain itself—through the TEE-protected acquisition and freshness mechanisms outlined in Section 6.3—is a complementary direction for moving from laboratory evaluation toward real-world deployment.

## 7. Conclusions

This work contributes a shift from evaluating power side-channel fingerprinting as a static identification problem to treating it as a temporally constrained intrusion detection problem. By defining and experimentally validating a unified design space, our results indicate that the key determinant of practical viability is not single-session separability but the ability of feature representations to maintain device identity under temporal variation.

We demonstrated that power side-channel fingerprinting combined with metric learning distinguishes batch-identical microcontrollers for IoT intrusion detection under the controlled laboratory conditions evaluated here. Evaluating seven ESP32 and seven Arduino devices over 45 days, we quantified manufacturing-induced electrical variations that produce discriminable power signatures despite identical hardware and firmware.

ESP32 achieved 1.80× higher separation than Arduino, though whether this reflects architectural complexity or other platform differences requires further investigation. Arduino showed stronger workload dependence (2.14× variation), while ESP32 maintained consistent separability.

Multi-session analysis exposed a critical limitation of handcrafted features. Under a matched LORO protocol, statistical features reached only 66.4% TPR at 35.8% FAR, while neural embeddings under the identical protocol and scoring rule achieved 93.9% TPR at 9.9% FAR—a 27.5 percentage point gain in true positive rate and a 25.9 point reduction in false acceptance. Ablation analysis identified AM-Softmax angular margin enforcement as the mechanism principally responsible for that gain. The advantage carries over when the impostor identity is withheld from training altogether: across 168 device-folds, embeddings reach 26.0% false acceptance against 39.0% for statistical features.

Statistical validation confirmed adequate sample size for proof-of-concept evaluation (89.1% power). These findings indicate that power-based verification is worth investigating as one factor within a multi-factor IoT security system.

Three directions will advance toward production deployment: extended temporal studies (6–12 months) in controlled environments, validation across larger multi-batch device populations, and adversarial robustness assessment including generative impersonation attacks. More broadly, integrating power side-channel fingerprinting with RF analysis, behavioral monitoring, and cryptographic protocols offers a promising path toward comprehensive defense-in-depth architectures addressing supply-chain vulnerabilities. While substantial development remains before critical infrastructure deployment, this framework provides an evaluation methodology and a proof-of-concept result on which practical IoT security systems could be built.

## Figures and Tables

**Table 1:** Comparison of Power- and Current-Based Device Fingerprinting Approaches, with MCU-Token Included as the Closest Microcontroller-Level Predecessor. A ✓ denotes that a dimension is addressed; the ✗ denotes that it is not.

Paper	Power	MCU	Same- Model	Open- Set	Temporal	Intrusion Det.
Spolaor et al. (2023) [10]	✓	✗	✓	✗	✗	✗
He and Sayadi (2025) [18]	✓	✗	✓	✗	✗	✗
Ronkin et al. (2022) [17]	✓	✗	✓	✗	✗	✗
Ronkin et al. (2023) [12]	✓	✗	✓	✗	✗	✗
Botvinnik et al. (2023) [11]	✓	✗	✓	✗	Limited ^‡^	✗
Myridakis et al. (2018) [19]	✓	✗	✗	✗	✗	✓
Park et al. (2020) [20]	✓	✗	✗	✗	✗	✓
Jung et al. (2022) [21]	✓	✗	✗	✗	✗	✓
Lightbody et al. (2024) [22]	✓	✗	✗	✗	✗	✓
Xiao et al. (2024) [2] ^†^	✗	✓	✓	✗	✗	✗
**This Work**	✓	✓	✓	✓	✓	✓

^†^ MCU-Token derives its fingerprint from on-chip peripheral responses (DAC/ADC, FPU, PWM, RTC, SRAM start-up) rather than from externally measured supply current, and is included here as the closest microcontroller-level hardware-fingerprinting predecessor. ^‡^ Botvinnik et al. repeat acquisition at gaps of 2–10 days and report substantial degradation over that interval; no evaluation beyond 10 days is reported, so the temporal evidence is partial rather than absent.

**Table 2:** Experimental Workloads and Targeted Hardware Components.

Task Name	Description	Hardware Component	Platform	Evaluation Type
Compute–Hash–Sleep	SHA-256 hashing, pseudo-random number generation (PRNG), and deep sleep cycles	ALU, SRAM, RTC	ESP32 & Arduino	Cross-platform & Same-platform
Bubble Sorting	Memory-intensive array sorting algorithm with repeated element swaps	ALU, SRAM, memory controller	Arduino	Same-platform
Networked Sensor Monitoring	Arithmetic operations combined with sensor data acquisition and Wi-Fi/Message Queuing Telemetry Transport (MQTT) connectivity	ALU, SRAM, GPIO, Wi-Fi RF	ESP32	Same-platform & Multi-run temporal

**Table 3:** Cross-Platform Baseline Performance Comparison (Day 0, Compute–Hash–Sleep).

Metric	ESP32	Arduino
Avg. Intra-device distance	0.323	0.437
Avg. Inter-device distance	0.988	0.740
Separation Ratio	3.06	1.70
d-prime ($d^{\prime }$)	2.54	1.25
Random Forest F1-Score	0.961	0.711

**Table 4:** Arduino Workload Complexity Impact on Device Discrimination (Day 0).

Metric	Compute–Hash–Sleep	Bubble Sorting
Avg. Intra-device distance	0.517	0.367
Avg. Inter-device Distance	0.995	1.505
Separation Ratio	1.92	4.10
d-prime ($d^{\prime }$)	1.58	3.18

**Table 5:** ESP32 Workload Consistency Analysis.

Metric	Hash-Sleep	Sensor Mon.
Avg. Intra-device dist.	0.346	0.321
Avg. Inter-device dist.	1.036	0.876
Separation Ratio	2.99	2.72
d-prime ($d^{\prime }$)	2.54	1.76

**Table 6:** ESP32 Classification Performance across Workloads (F1-Score).

Model	Hash-Sleep	Sensor Mon.
Logistic Regression	0.887	0.831
SVM (RBF)	0.914	0.819
Random Forest	0.955	0.962
KNN	0.907	0.820
Naive Bayes	0.874	0.806
Decision Tree	0.905	0.906

**Table 7:** Open-Set Intrusion Detection Performance: Raw Features vs. Neural Embeddings.

Protocol	Features	TPR	Real	Adv.	HW
			FAR	FAR	FAR
Fixed Split	Raw	72.3%	38.7%	49.7%	38.7%
Fixed Split	Embed.	94.6%	5.1%	15.8%	5.1%
CV Split	Raw	53.9%	20.1%	26.5%	19.7%
CV Split	Embed.	95.0%	4.7%	16.3%	4.8%
LORO Cross	Raw	66.4% [55.4, 77.1]	35.8% [25.8, 46.1]	43.7%	36.0%
LORO Cross	Embed.	93.9% [88.9, 97.9]	9.9% [5.2, 15.1]	26.8%	9.9%

TPR = True Positive Rate; FAR = False Acceptance Rate; Adv. = Adversary attack; HW = Hardware attack; Embed. = Embeddings.

**Table 8:** Confidence Intervals for Open-Set Detection Metrics (EER Threshold).

Protocol/Features	Metric	Mean	95% CI	n
Raw (Fixed, EER)	TPR	0.723	[0.580, 0.830]	7
	FAR_real_	0.387	[0.277, 0.478]	7
Embeddings (Fixed, EER)	TPR	0.946	[0.902, 0.991]	7
	FAR_real_	0.051	[0.022, 0.080]	7
Raw (CV, EER)	TPR	0.539	[0.436, 0.643]	35
	FAR_real_	0.201	[0.165, 0.238]	35
Embeddings (CV, EER)	TPR	0.950	[0.918, 0.979]	35
	FAR_real_	0.047	[0.029, 0.068]	35
Raw (LORO, EER)	TPR	0.664	[0.554, 0.771]	28
	FAR_real_	0.358	[0.258, 0.461]	28
Embeddings (LORO, EER)	TPR	0.939	[0.889, 0.979]	28
	FAR_real_	0.099	[0.052, 0.151]	28
Raw (LODO, EER)	TPR	0.663	[0.619, 0.709]	168
	FAR_unseen_	0.390	[0.324, 0.457]	168
Embeddings (LODO, EER)	TPR	0.911	[0.880, 0.940]	168
	FAR_unseen_	0.260	[0.207, 0.317]	168

LODO = leave-one-device-out; impostors are devices withheld from training entirely.

**Table 9:** Ablation Study Results (LORO cross-session, EER Threshold).

Configuration	TPR	FAR	TPR Delta	Impact
Raw features (no network)	66.4%	35.8%	−27.9	—
Full model	94.3%	11.7%	0.0	—
Triplet loss only	81.8%	15.8%	−12.5	Critical
AM-Softmax only	94.3%	11.3%	0.0	None
No residual connection	89.3%	9.6%	−5.0	Moderate
No batch normalization	89.6%	9.8%	−4.6	Moderate
No data augmentation	94.3%	12.0%	0.0	None
Euclidean scoring	93.9%	11.7%	−0.4	None
32D embeddings	93.6%	10.7%	−0.7	None
128D embeddings	91.8%	9.3%	−2.5	Moderate

**Table 10:** Projected Deployment Memory Footprint (Model + Centroid Gallery).

Enrolled Devices	Float32 (KB)	Int8 (KB)
7	310.0	78.8
50	320.8	89.6
100	333.2	102.1
500	433.2	202.1
1000	558.2	327.1
5000	1558.2	1327.1

ESP32 SRAM: 520 KB; Flash: 4096 KB. Flash storage only; runtime arena is 64 KB SRAM (Section 5.6).

**Table 11:** Inference Latency vs. Enrolled Device Count (CPU Baseline, x86_64).

Gallery Size	Embed. (ms)	Scoring (μs)
7	0.169±0.020	23.0±17.7
50	0.169±0.020	33.5±19.1
100	0.169±0.020	37.9±3.1
500	0.169±0.020	103.8±16.5
1000	0.169±0.020	210.5±55.7
5000	0.169±0.020	1071.3 ± 180.0

Embedding timed in PyTorch (CPU); gallery scoring timed in NumPy (CPU). 500 trials; 10 warm-up passes excluded. Mean ± std reported.

**Table 12:** Perturbation-Based Minimum Angular Separation by Embedding Dimension.

Dimension	Min. Sep.	Min. Sep.	Parameters
	at n=7	at n=5000	(k)
16D	77.8°	15.2°	72.8
32D	74.9°	23.6°	75.0
64D	73.8°	29.9°	79.4
128D	76.2°	33.9°	88.1
256D	75.2°	36.9°	105.5

All dimensions achieve >73° at 7 devices. Higher dimensions sustain larger minimum separation at scale. Angle thresholds are geometric observations, not validated performance boundaries. Parameter counts are totals including the AM-Softmax head; the inference-only 64D encoder is 78,912 (Section 5.6).

## Data Availability

The power-trace recordings and derived feature matrices supporting the reported results are available from the corresponding author on reasonable request. The firmware and analysis scripts are not yet released, as they require further cleanup and documentation to be usable as a package rather than a set of working scripts. The parameters needed to reproduce the measurement and analysis pipeline are specified in the manuscript: the workloads and their targeted subsystems (Section 4.3, Table 2), the window-extraction thresholds and normalization protocol (Section 4.4), the statistical feature set and its three-stage screening (Section 3.2), the embedding architecture and training configuration (Section 3.4.3), the three thresholding strategies and the three evaluation protocols (the same section), and the random seed used throughout.

## Abbreviations

The following abbreviations are used in this manuscript:

ADC	Analog-to-Digital Converter
AM-Softmax	Additive-Margin Softmax
CNN	Convolutional Neural Network
COTS	Commercial Off-The-Shelf
DDoS	Distributed Denial-of-Service
DoS	Denial-of-Service
DT	Decision Tree
DVFS	Dynamic Voltage and Frequency Scaling
EER	Equal Error Rate
FAR	False Acceptance Rate
FFT	Fast Fourier Transform
FPR	False Positive Rate
FRR	False Rejection Rate
GNB	Gaussian Naive Bayes
IDS	Intrusion Detection System
IoT	Internet of Things
K-NN	K-Nearest Neighbors
LODO	Leave-One-Device-Out
LORO	Leave-One-Run-Out
LR	Logistic Regression
MAC	Message Authentication Code
MCU	Microcontroller Unit
MQTT	Message Queuing Telemetry Transport
PCA	Principal Component Analysis
PKI	Public Key Infrastructure
PUF	Physical Unclonable Function
RF	Radio Frequency
RFC	Random Forest Classifier
SCADA	Supervisory Control and Data Acquisition
SDR	Software-Defined Radio
SVDD	Support Vector Data Description
SVM	Support Vector Machine
TPR	True Positive Rate
